# Extracellular Vesicles of *Giardia duodenalis*: Unravelling Their Virulence Factors and Potential to Induce Protection Against Experimental Giardiasis

**DOI:** 10.1002/jex2.70155

**Published:** 2026-06-11

**Authors:** Corral‐Ruiz Gerardo, Argüello‐García Raúl, Ortiz‐Lozano Dalia, Salazar‐Villatoro Lizbeth, Pech‐Santiago Edar Onam, Espinosa‐Cantellano Martha, Hernández‐Cueto Daniel, Silva‐Olivares D. Angélica, Betanzos Abigail, Ortega‐Pierres M. Guadalupe

**Affiliations:** ^1^ Departamento de Genética y Biología Molecular Centro de Investigación y de Estudios Avanzados del Instituto Politécnico Nacional Mexico City Mexico; ^2^ Departamento de Infectómica y Patogénesis Molecular Centro de Investigación y de Estudios Avanzados del Instituto Politécnico Nacional Mexico City Mexico; ^3^ Unidad de Investigación en Enfermedades Hemato‐Oncológicas Hospital Infantil de México Federico Gómez Mexico City Mexico

**Keywords:** epithelial cell damage, extracellular vesicles, giardia duodenalis, mucosal immunization, virulence factors

## Abstract

Extracellular vesicles (EVs) are key mediators of intercellular communication, enabling trans‐kingdom exchange of proteins, nucleic acids, lipids, and metabolites. In *Giardia duodenalis*, EVs contribute to parasite survival, host–pathogen interactions, and immune modulation. In this study, EVs were isolated from trophozoites grown in two serum‐free media of distinct nutritional complexity (TYI‐33 and DMEM) and characterized to define their molecular composition, pathogenic potential, and immunogenic properties. Nanoparticle tracking analysis showed vesicles predominantly 51–150 nm in diameter. Proteomic profiling identified 227 proteins in TYI‐33 EVs and 97 in DMEM EVs, including molecules involved in metabolism, antioxidant defence, and virulence. Among these, giardipains 1–3, enolase, fructose‐bisphosphate aldolase, ornithine carbamoyltransferase, and arginine deiminase were detected. TYI‐33 EVs induced disruption of epithelial junctions and apoptotic‐like changes in IEC‐6 monolayers, consistent with contact‐independent cytopathic activity. To assess their immunoprotective capacity, gerbils were intranasally immunized with TYI‐33 EVs and challenged with *G. duodenalis*. Immunized animals showed significant reductions in intestinal trophozoite burden and faecal cyst shedding at 7, 14, and 21 days post‐infection, accompanied by increased serum IL‐17F and IL‐22. Collectively, these findings indicate that *Giardia* EVs, while carrying virulence‐associated factors, also possess strong immunogenic properties, supporting their potential as a novel mucosal vaccine platform against giardiasis.

## Graphical abstract

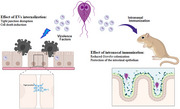

## 1 Introduction

Human giardiasis is a worldwide parasitic disease caused by the flagellated protozoan *Giardia duodenalis* (syn. *G. lamblia*, *G. intestinalis*). Annually, 280 million new cases are estimated at a prevalence of 2%–5% and 20%–30% in developed and developing countries respectively (Savioli et al. 2006). The life cycle of *Giardia* alternates between a cyst form, highly resistant to environmental stress, and the trophozoite, which is the active and motile stage responsible for colonizing the small intestine (Ankarklev et al. 2010). Human infection occurs by the faecal‐oral route after ingestion of food or water contaminated with cysts. Most infections are usually asymptomatic and self‐limiting, that typically resolve within few weeks in immunocompetent individuals, while symptomatic infections are often accompanied by diarrhoea, abdominal pain, malabsorption, and weight loss (Robertson et al. 2010).

*Giardia* has evolved several strategies to evade host immunity, contributing to chronic infection and the persistence of symptoms in certain individuals (Robertson et al. 2010; Argüello‐García and Ortega‐Pierres 2021; Argüello‐García et al. 2023; Solaymani‐Mohammadi 2022). Among these mechanisms are the secretion of extracellular vesicles (EVs) by the parasite, which mainly includes large extracellular vesicles (LEVs) and small extracellular vesicles (sEVs) (Sana et al. 2023). EVs are essential for cellular communication, allowing trans‐kingdom interactions and mediating the transfer of proteins, nucleic acids, lipids, and metabolites (Mardahl et al. 2019; Carrera‐Bravo et al. 2021). They enable constant crosstalk among species and have a crucial role in developing different biological functions (Moccia et al. 2022; Sharma et al. 2023). Recent studies highlighted the involvement of *Giardia* EVs in parasite survival, pathophysiology and modulation of host's immune response, making them a key element in the pathogenesis of giardiasis (Evans‐Osses et al. 2017; Zhao et al. 2021a; Zhao et al. 2021b; Natali et al. 2023).

To date, the precise pathogenic mechanisms underlying giardiasis are not fully understood. Pathogenesis is closely related to interactions between trophozoites and host intestinal epithelial cells (Ortega‐Pierres and Argüello‐García 2019; Argüello‐García et al. 2023), along with the release of multiple *Giardia*‐derived molecules, including virulence factors such as metabolic enzymes as enolase (Barroeta‐Echegaray et al. 2022) and proteases (i.e., cathepsins B) like giardipain‐1, which have been identified in EVs (Ma'ayeh et al. 2017). These factors, secreted in a soluble form, are capable of inducing damage to intestinal epithelial cells, leading to apoptotic cell death and disruption of intercellular tight junctions (Ortega‐Pierres et al. 2018; Allain et al. 2019; Barroeta‐Echegaray et al. 2022). This process also promotes the translocation of bacterial and parasite‐derived products, resulting in an inflammatory state in the infected host (Beatty et al. 2017).

Characterization of *Giardia* EVs has revealed a complex lipid composition, which may be crucial to their functionality, potentially enhancing interactions with host cell membranes and promoting vesicle internalization (Faria et al. 2023). Exposure of human intestinal epithelial cells (CaCo‐2) to *Giardia* excretory‐secretory products (ESPs) containing EVs led to differential gene expression, influencing their functional response (Ma'ayeh et al. 2017; Yang et al. 2024). Moreover, *Giardia* EVs interact not only with epithelial cells but also with immune cells such as macrophages, influencing immune signalling pathways. These vesicles can be recognized by the immune system, inducing the activation of Toll‐like receptors (TLRs) and inflammasomes like NLRP3 (Zhao et al. 2021a). These interactions can trigger the production of pro‐inflammatory cytokines, contributing to the inflammatory response and tissue damage commonly observed in infected individuals (Evans‐Osses et al. 2017; Zhao et al. 2021a; Zhao et al. 2021b). Furthermore, the effects of giardial EVs extend beyond their impact on host cells, as they also influence the composition and function of the intestinal microbiome, altering gut homeostasis (Siddiq et al. 2023). Moreover, *Giardia* EVs appear to enhance the parasite's adhesive capacity, potentially facilitating its persistence within the host (Gavinho et al. 2020).

The versatility of EVs, combined with their capacity to cross biological barriers, make them highly promising candidates for various biomedical applications (Takahashi and Takakura 2023; Kumar et al. 2024). EVs production and composition are known to be influenced by the culture environment such as medium composition, serum supplementation and culture density, indeed the use of serum‐free or chemically defined media is recommended to reduce variability (Fernandez‐Becerra et al. 2023). In *Giardia duodenalis*, environmental conditions also appear to modulate EV content. Recent proteomic analyses have shown that stimuli such as bile exposure alter the protein composition of *Giardia*‐derived EVs, suggesting that culture conditions may directly affect their biological function and host–pathogen interactions (Siddiq et al. 2023). Ongoing research is focused on harnessing their unique properties to develop advanced diagnostic tools and novel therapeutic strategies (Vidal et al. 2024). For instance, in a colitis model induced by dextran sodium sulfate (DSS), *Giardia* EVs have been shown to reduce the inflammatory damage, while the potential of EVs to be used as vaccine has been validated in the *Neisseria meningitidis* model (Kim et al. 2022, Alfandari et al. 2023; Castilla et al. 2023). Since EVs represent a complex antigenic mosaic and are continuously released during host‐parasite interactions, they could be exploited for their ability to induce protective immunity against giardiasis (Alfandari et al. 2023). Therefore, in this study, we characterized EVs derived from *G. duodenalis* WB trophozoites and assessed their involvement in contact‐independent pathogenic mechanisms. Importantly, we also compared EVs obtained from two serum‐free culture media of distinct nutritional complexity (TYI‐33 and DMEM) to evaluate how the growth environment influences EV composition and potential immunomodulatory properties.

## 2 Material and Methods

### 2.1 Ethics Statement

All procedures with animals were conducted in strict accordance with the guidelines and regulations of the Committee for the Care and Use of Laboratory Animals (CICUAL) at Cinvestav, Mexico City, Mexico (Protocol #0165‐15). A total of 124 gerbils (8–10 weeks old) were housed under controlled 12‐h light/dark cycles with sterilized bedding and environmental enrichment and were provided ad libitum access to sterilized water and a commercial diet. All handling, breeding, and experimental procedures were performed by trained and experienced personnel to ensure animal welfare.

### 2.2 Parasites

Trophozoites of *G. duodenalis* WB strain (Assemblage A ATCC # 30957) maintained in cryopreservation in liquid nitrogen were reactivated by thawing vials and cultivated at 37°C using Keister's modified TYI‐S‐33 medium supplemented with 10% bovine serum (Hyclone, South Logan, USA), and 1% antibiotic/antimycotic mixture (Keister. 1983). Also, we used trophozoites maintained for 4 h at 37°C in Dulbecco´s Modified Eagle Medium (DMEM) with added glutamate [4 mM] and lacking bovine serum (Evans‐Osses et al. 2017; Gavinho et al. 2020; Zhao et al. 2021a).

### 2.3 Isolation of EVs

EVs from *G. duodenalis* trophozoites were collected from confluent culture tubes via stepwise centrifugation. First, dead cells and unattached parasites were removed from the *G. duodenalis* cultures. TYI‐33 medium was added without bovine serum to avoid contamination from serum‐derived exosomes and incubated for 4 h at 37°C to allow EV secretion, with [1 mM] CaCl_2_ added as an inducer of EV release. Also, EVs were collected from trophozoites maintained for 4 h at 37°C in DMEM with added glutamate [4 mM] and with no bovine serum (Ma'ayeh et al. 2017; Grajeda et al. 2022). At the end of the incubation, trophozoite viability was assessed by trypan blue exclusion, which was over 99% (Fernandez‐Becerra et al. 2023). The two sets of EVs obtained as aforementioned were named TYI‐33‐EVs and DMEM‐EVs respectively.

Isolation of *Giardia* EVs was carried out by multiple differential centrifugations. To eliminate parasites and cellular debris, low speed centrifugations were done at 300 x *g*, 600 x *g* and 2000 x *g* for 15 min each at 4°C. Then, the supernatant was subjected to centrifugation at 15000 x *g* for 30 min at 4°C to remove large vesicles. Finally, the remaining supernatants were ultracentrifuged using the 70Ti rotor (Beckman Coulter, California, USA) at 100,000 x *g* for 2 h at 4°C. Pellets enriched in sEVs were resuspended in PBS, and protein content was quantified using a BCA Protein Assay Kit. (Thermo Scientific, Massachusets, USA). TYI‐33–derived EVs were used for nanoparticle tracking analysis, transmission electron microscopy, proteomic profiling, western blotting, EV internalization assays, immunofluorescence studies, scanning electron microscopy, and gerbil immunization experiments. DMEM‐derived EVs were used for nanoparticle tracking analysis, transmission electron microscopy, proteomic profiling, and scanning electron microscopy.

### 2.4 Nanoparticle Tracking Analysis

EVs sizes were measured using a NanoSight NS300 (Malvern Panalytical Ltd., Malvern, UK) apparatus. Samples were diluted 1:10,000 in filtered PBS. Each sample (five technical repeats) was recorded for 60s using the following parameters: camera level 13; slider shutter 1232; slider gain 219; and 25 FPS. The computational analysis was performed using NTA v3.00 software.

### 2.5 Transmission Electron Microscopy

EVs were observed by negative staining, following the standard method. Secreted EVs (5 µL) were pipetted onto the surface of Formvar‐coated copper grids (400 mesh). The samples were dried with filter paper and stained with 2.5% uranyl acetate for 20 s. Grids were air‐dried and carbon‐coated in a vacuum evaporator (JEE400, JEOL Ltd., Tokyo, Japan). The samples were examined using a JEM‐1011 transmission electron microscope (Castelan‐Ramírez et al. 2025).

### 2.6 Label‐Free Proteomic Analysis of *Giardia* EVs

Purified EVs pellets from trophozoites grown in TYI‐33 or DMEM were lysed in RIPA buffer (10 mM Tris−HCl pH 8.0, 1 mM EDTA, 1% Triton X‐100, 0.1% sodium deoxycholate, 0.1% SDS, 140 mM NaCl and complete mini EDTA‐free protease inhibitor cocktail [Roche^TM^]) for 30 min at 4°C with gentle shaking. For label‐free quantification, input amounts were set at 50 µg per sample with further digestion with trypsin (Promega) (1:100 protein ratio) at 37°C overnight. Following tryptic digestion, peptides were first captured on a Symmetry C18 Trap V/M precolumn (Waters), enabling on‐line desalting and removal of residual detergents and contaminants; afterwards digestion products were resuspended in 0.1% formic acid prior to analytical separation on an HSS T3 C18 column coupled to tandem mass spectrometer (LC‐MS) to identify peptides by a label‐free protocol as described elsewhere (Ríos‐Castro et al. 2020). MS measurements contained in the generated *.raw files were normalized, aligned, identified (Li et al. 2009), compared, and relatively quantified using Progenesis QI for Proteomics software v3.0.3 (Waters, Milford, MA) (Geromanos et al. 2011; Valentine et al. 2011) against a *Giardia intestinalis* database, (strain ATCC 50803, downloaded from Uniprot release UP000001548, containing 4900 protein sequences). Parameters included: trypsin enzyme, one missed cleavage allowed, carbamidomethyl (C) as a fixed modification with oxidation (M), phosphoryl (S, T, Y) as variable modifications, peptide and fragment tolerance at maximum normal distribution of 10 and 20 ppm respectively and false discovery rate (FDR) ≤4%. The latter threshold was chosen to allow more robust peptide spectra, not only to enhance detection of low‐abundance peptides with higher coincidence to previous studies but aiming a better relationship between the known biological function of proteins identified herein and results obtained in other studies. Results from Progenesis software were exported to *.xlsx files to verify the levels of reliability and confidence for label‐free experiments (peptide and protein levels) according to the figures of merit (FOM) described by Souza et al. (2017). For manual validation of data analyses, identified duplicates and contaminants were excluded along with low score spectra from the list of each sample, thereby all filtered proteins were considered. The Gene Ontology (GO) database was generated through the PANTHER tool (https://pantherdb.org/). Protein‐protein interactions in cargo proteins within EV samples were analyzed using the STRING database (https://string‐db.org/).

### 2.7 Western Blot Analysis to Detect Giardipain‐1, Enolase, and Variant‐specific Surface Protein (VSP) Present in G. *duodenalis* EVs

Freshly isolated EVs were lysed in RIPA buffer, and 30 µg of total protein was separated by electrophoresis on 12% polyacrylamide gels under denaturing conditions (SDS–PAGE). Proteins were subsequently transferred onto nitrocellulose membranes at 300 mA for 1 h under cold conditions. Membranes were blocked with 5% low‐fat milk in 0.1% Tween (TBS‐T) for 1 h at room temperature (RT) under constant shaking. After blocking, membranes were washed three times in 0.1% TBS‐T and incubated overnight at 4°C, under constant agitation, with the following primary antibodies diluted in 5% low‐fat milk/TBS‐T: anti‐giardipain‐1 (IG3A11 monoclonal; 1:500), anti‐enolase (polyclonal; 1:250), and anti‐variant‐specific surface protein (VSP9B10A; polyclonal; 1:100) (Barroeta‐Echegaray et al. 2022., Cabrera‐Licona et al. 2017; Hernández‐Sánchez et al. 2008). Following incubation, unbound primary antibodies were removed by three consecutive washes in 0.1% TBS‐T. Membranes were then incubated with horseradish peroxidase (HRP)‐conjugated anti‐mouse immunoglobulins (GE Healthcare, Pittsburgh, PA, USA) at a 1:20,000 dilution in 5% low‐fat milk/TBS‐T for 1 h at RT under constant shaking. Immunoreactive bands were visualized using the ECL Plus Lightning Western Blotting Detection Kit (PerkinElmer, Waltham, MA, USA).

### 2.8 Interaction of EVs With IEC‐6 Cell Monolayers

IEC‐6 cell line (ATCC Cat. No. CRL1592) was seeded on coverslips in the bottom of 24‐well plates (Sigma, Hamburg, Germany) and cultured in DMEM medium (GibcoBRL, Gaithersburg, MD, USA) supplemented with 10% FBS (HyClone, Marlborough, MA, USA), 0.1% glutamine and 1% antibiotic/antimycotic mixture (HyClone, Marlborough, MA, USA) at 37°C and 5% CO_2_. Confluent cell monolayers were incubated in FBS‐free DMEM with freshly obtained EVs equivalent to 100 µg of total protein for 3 h or with 2 × 10^6^ trophozoites as a control under the conditions mentioned before. Samples were washed with PBS and analyzed by confocal and scanning electron microscopy.

### 2.9 EVs Internalization by IEC‐6 Cells

Freshly isolated TYI‐33‐EVs were stained with Dil fluorophore (Thermo Scientific, Massachusetts, USA) to label the EV membrane and Syto RNA (Thermo Scientific, Massachusetts, USA) to stain the EV RNA cargo. Additionally, the EVs cargo was also stained with CFSE, a fluorophore that labels amine groups at the protein N‐ terminus, particularly aspartate and glutamate residues (Chen et al. 2009). Once labelled, the EVs were concentrated overnight using the Total Exosome Isolation Reagent (Thermo Scientific, Massachusetts, USA). The sample was then centrifuged at 10,000 x *g* for 1 h at 4°C. The pellet was resuspended in PBS, and EVs equivalent to 100 µg of total protein were incubated with confluent IEC‐6 cell monolayers in FBS‐free DMEM for 3 h at 37°C. Following incubation, samples were analyzed by flow cytometry using a FACS Calibur (Becton Dickinson, New Jersey, USA) and the FlowJo v11 software. For fluorescence microscopy, the samples were counterstained with DAPI, examined using a Leica SP8 microscope (Wetzlar, Germany) and analyzed using LAS X v5.1 software.

### 2.10 Immunofluorescence Assays

IEC‐6 cell monolayers were washed with PBS containing Mg^2+^ and Ca^2+^ and fixed with cold acetone for 5 min and ethanol for 1 min. For non‐specific blocking, 1% (*v*/*v*) BSA in PBS was used for 1 h.

Subsequently, rabbit anti‐ZO‐1 primary antibody diluted 1:100 (Cat. SAB5700645, Invitrogen, Camarillo, CA, USA) was added and incubated overnight at 4°C. After three washes with PBS, secondary anti‐rabbit Igs coupled to Alexa 647 and phalloidin coupled to Alexa Fluor 488 (Cat. A12379, Invitrogen Camarillo, CA, USA), were added and incubated for 2 h at RT. Again, three washes with PBS were performed and preparations were incubated for 10 min with 2.5 µg/ml 4',6‐diamino‐2‐phenylindole (DAPI; Cat. D9542, Sigma, Hamburg, Germany) (Betanzos et al. 2013). Samples were washed and mounted with Vectashield (Cat. H‐1000‐10, Vector, Torrance, CA, USA), then analyzed with a Carl Zeiss LMS 900 confocal microscope (Oberkochen, Germany, Zeiss) in Z‐stack optical sections of 0.5 µm and *xz*‐ and *zy*‐planes and processed with ZEN 2009 Light Edition Software (Oberkochen, Germany, Zeiss).

### 2.11 Scanning Electron Microscopy

For scanning electron microscopy, PBS‐washed samples were fixed with 2.5% glutaraldehyde in 0.1 M sodium cacodylate buffer, pH 7.2, dehydrated under increasing concentrations of ethanol and hexamethyldisilazane (HMDS) to remove liquids from the biological specimen. Afterwards samples were gold‐coated in an ion‐sputtering device (JOEL‐JFC‐1100, Jeol USA Inc., Peabody, MA, USA). Samples were analyzed in a JSM‐7100F scanning electron microscope (Ortega‐Pierres et al. 2018).

### 2.12 Gerbil Immunization and Infection

Mongolian gerbils (*Meriones unguiculatus*) were immunized intranasally with TYI‐33‐EVs (10 µg total protein in 10 µL volume) into a single nostril. Immunizations were performed three times once a week. Infection challenges were conducted two weeks after the last immunization. For infection experiments, tubes with at least 90% of trophozoite confluence were used. Dead parasites were removed from the culture medium, and trophozoites were detached by placing the tube on ice for 30 min. Parasites were collected by centrifugation (750 x *g* for 10 min at 4°C) and washed once with PBS. Parasites were counted using a haemocytometer and diluted to the appropriate concentration. Gerbils from control (non‐immunized) and experimental (immunized) groups were infected by oral gavage with 1 × 10^6^ trophozoites suspended in a 500 µL volume in PBS.

### 2.13 Quantification of Trophozoites and Cysts of G. *duodenalis* in Infected Gerbils

Infected gerbils (control and immunized) were euthanized at different times post‐infection. The small intestine was excised and opened longitudinally in cold PBS. To collect the trophozoites from the intestinal mucosa, the tissues were placed in PBS under constant shaking for 60 min. The suspension was centrifuged at 750 x *g* for 10 min at 4°C, the pellet was resuspended in 100 µL PBS, and the number of parasites was determined by counting collected trophozoites using a hemacytometer (Hernández‐Sánchez et al. 2008).

For quantification of cyst excretion, faecal samples obtained over a 24 h period were homogenized, washed with PBS by centrifugation, resuspended in 33% ZnSO_4_ solution, and centrifuged at 650 x *g* for 5 min. The supernatant layer was collected and diluted in double‐distilled water and centrifuged under the same conditions. The pellet was resuspended in 100 µL double‐distilled water, and cysts were counted in a haemocytometer (Hernández‐Sánchez et al. 2008).

### 2.14 Serum Cytokine Quantification

Whole blood was collected from infected gerbils (control and immunized) by cardiac puncture, sera were obtained and stored at −80°C until analysis. Serum concentrations of IL‐17F and IL‐22 were quantified using the LEGENDplex Mouse Th17 Panel (BioLegend, San Diego, CA, USA), following the manufacturer's protocol. Briefly, cytokine standards and serum samples were incubated in the dark at RT with a mixture of capture beads for 2 h. Subsequently, detection antibodies were added and incubated for 1 h, followed by a 30‐min incubation with Streptavidin‐Phycoerythrin (SA‐PE). After washing, samples were resuspended in assay buffer and analyzed by flow cytometry using a FACSAria III instrument (BD Biosciences, New Jersey, USA). Data acquisition was performed with the LEGENDplex Data Analysis Software Suite. Standard curves were generated using log‐transformed values and a five‐parameter logistic (5‐PL) regression model, and cytokine concentrations in the samples were interpolated accordingly.

### 2.15 Histopathological Analysis

Infected gerbils from both control and immunized groups were euthanized at 7, 14, and 21 days post‐infection (dpi). Following euthanasia, the duodenum was carefully dissected, rinsed with PBS to remove intestinal contents, and fixed in 10% neutral‐buffered formalin for 24 h at room temperature. Fixed tissues were then processed through graded alcohols, cleared in xylene, and embedded in paraffin. Paraffin blocks were sectioned at a thickness of 4 µm using a rotary microtome, and the sections were mounted on glass slides. For histopathological analysis, the sections were deparaffinized, rehydrated, and stained with haematoxylin and eosin (H&E) following standard procedures (Quezada‐Lázaro et al. 2022). Images were acquired using an Axiocam MRc digital camera mounted on an Axio Scope.A1 microscope (Zeiss, Oberkochen, Germany), and representative fields were analyzed for morphological alterations.

### 2.16 Statistical Analysis

Statistical analyses were carried out using SigmaPlot version 12.0. Comparisons between groups were performed using the Mann–Whitney U test. Results are presented as mean ± SEM, and differences were considered statistically significant at *p* ≤ 0.05 (*p ≤ 0.05, **p ≤ 0.01, ***p ≤ 0.001).

## 3 Results

### 3.1 Structural Characterization of G. *duodenalis* EVs

**FIGURE 1:**
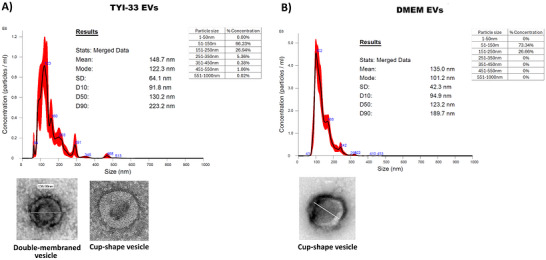
**Structural characterization of *Giardia duodenalis* EVs**. **A)** TYI‐33‐EVs and **B)** DMEM‐EVs particle size distribution analyzed by NTA, and morphological characterization performed by TEM using negative staining. The profile shows a heterogeneous population of vesicles, with a predominant peak within the size range for exosome‐like vesicles (51–150 nm). Images reveal round, membrane‐bound vesicles with typical cup‐shaped morphology and sizes consistent with those observed by NTA.

To stimulate EVs production, *G. duodenalis*, trophozoites were exposed to CaCl_2_. Using nanoparticle tracking analysis (NTA), we found that TYI‐33‐derived EVs had a mean diameter of 148.7 nm, with a modal peak at 122.3 nm. Size distribution analysis showed that most vesicles (66%) fell within the 51–150 nm range. In comparison, DMEM‐derived EVs had a mean diameter of 135 nm, with a modal peak at 101.2 nm, and 73% of vesicles were also distributed within the 51–150 nm range. Overall, these profiles are consistent with the typical size range reported for sEVs (Figure 1A). Importantly, EVs obtained from both media exhibited comparable morphology. Transmission electron microscopy (TEM) with negative staining revealed a heterogeneous population of membrane‐bound vesicles displaying both spherical and cup‐shaped morphologies (Figure 1B). These structural characteristics are consistent with *Giardia‐*derived EVs described by other groups (Faria et al. 2023; Natali et al. 2023 Siddiq et al. 2023).

### 3.2 Proteomic Analyses of G. duodenalis EVs and Detection of Virulence Factors

**FIGURE 2:**
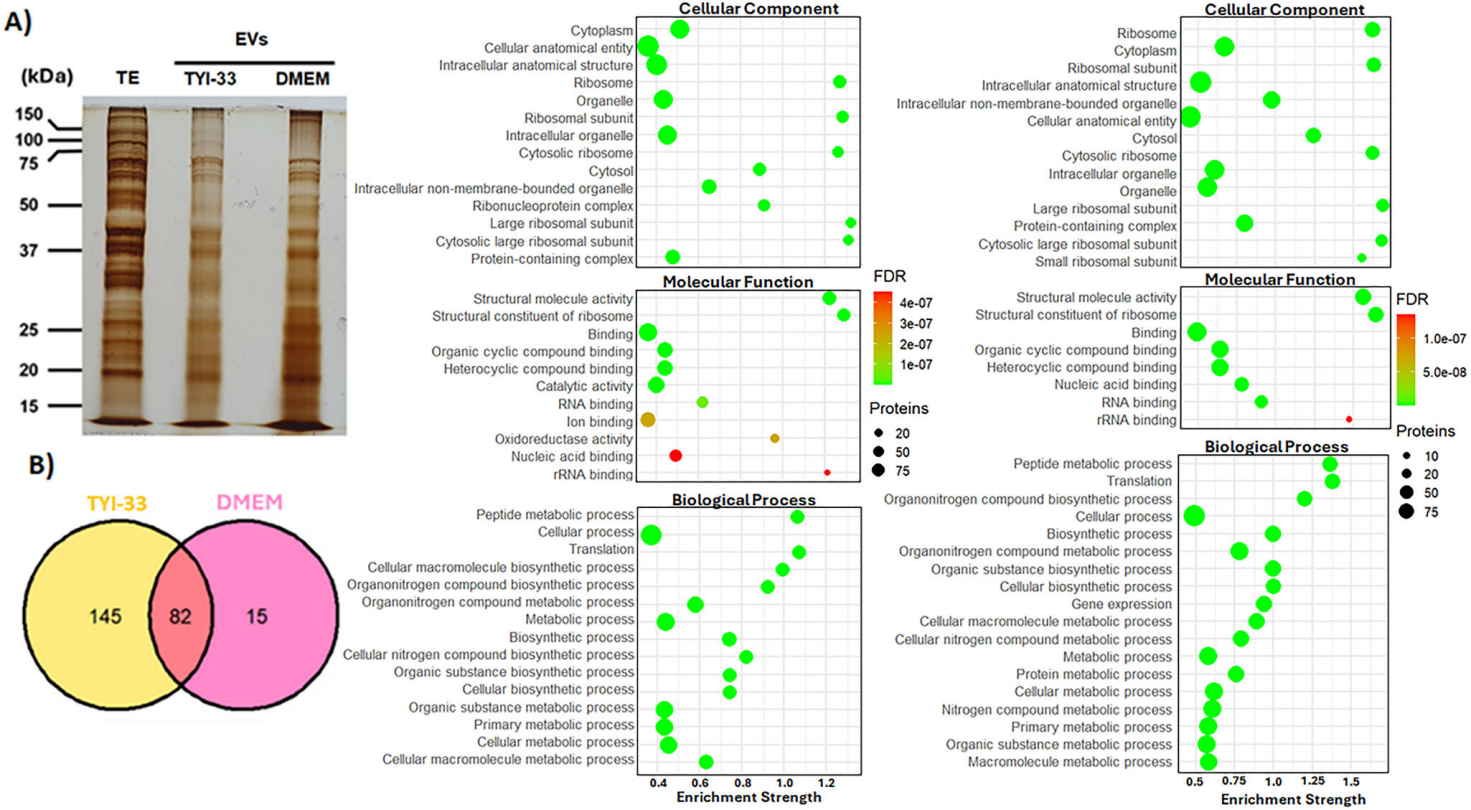
**Proteomic analyses of *G. duodenalis* EVs derived from TYI‐33 and DMEM cultured trophozoites. A)** SDS‐PAGE analysis of EVs; TE, Trophozoite extract. **B)** Venn diagram of proteins identified through LC‐MS analysis in EVs derived from TYI‐33 and DMEM cultured trophozoites. GO analysis of proteins identified in **C)** TYI‐33‐EVs and **D)** DMEM‐EVs.

With the aim of assessing potential differences in protein composition, EVs isolated from trophozoites cultured in TYI‐33 and in DMEM media were analyzed by polyacrylamide gel electrophoresis followed by silver staining. Both EV preparations shared several prominent high‐ (>65 kDa) and low‐ (22–23 kDa) molecular‐weight bands. However, distinct protein bands were observed between 25 and 60 kDa, indicating media‐dependent variations in EV cargo composition. Figure 2A shows the electrophoretic pattern of EVs obtained from TYI‐33 and DMEM, displaying the protein bands detected under each condition.

Subsequently, label‐free LC‐MS analyses were conducted to profile the protein content of *G. duodenalis* EVs isolated from trophozoites cultured in either TYI‐33 or DMEM. A total of 6,807 peptides corresponding to 242 distinct proteins were identified. Among these, 82 proteins were common to both EV preparations, while 145 were uniquely detected in TYI‐33‐EVs and 15 were exclusive to DMEM‐EVs (Figure 2B). Several canonical EV markers were identified, including two vesicle‐fusing ATPases (shared), as well as heat shock protein 90, vacuolar ATP synthase, and two vacuolar sorting proteins found exclusively in TYI‐33‐EVs. The most prominent shared parasite‐derived proteins included: (a) metabolic enzymes from arginine catabolism (arginine deiminase, ornithine carbamoyltransferase, and carbamate kinase) and glycolysis (enolase, fructose‐bisphosphate aldolase, and pyruvate‐phosphate dikinase); (b) cytoskeletal and motor proteins, including several giardins (α1, α7.1, α7.2, α7.3, α14), tubulins (α and β), and kinesins (types 2 and 3); and (c) enzymes involved in redox homeostasis, such as A‐type flavoprotein, peroxiredoxins 1 and 2, and NADH oxidase (Table 1).

**TABLE 1:** Functional classification of proteins identified by LC‐MS in EVs from *G. duodenalis* trophozoites cultured in TYI‐33 or DMEM. The peptide score, orthologue presence in other species and rank detection in top 100 proteins (Vesiclepedia^a^) is shown.

ORF	Name	TYI‐33	DMEM	Homologues in other (EV proteomes)	Rank in Vesiclepedia
Glycolysis					
GL50803_11118	Enolase	2704.8	1930.4	Yes (30)	14
GL50803_11043	Fructose‐bisphosphate aldolase	1223.3	869.8	Yes (5)	26
GL50803_9909	Pyruvate‐phosphate dikinase	2526.1	2380.6	No	—
GL50803_17143	Pyruvate kinase	223.8	na^b^	Yes (17)	15
GL50803_6687	Glyceraldehyde‐3‐phosphate dehydrogenase	995.5	na	Yes (23)	6
Arginolysis					
GL50803_112103	Arginine deiminase	2474.2	3897.2	Yes (1)	—
GL50803_10311	Ornithine carbamoyltransferase	6194.8	3713.8	Yes (3)	—
GL50803_16453	Carbamate kinase	1294.1	1266.6	No	—
Cytoskeletal and motor proteins					
GL50803_101291	Tubulin beta chain	12064.0	11919.2	Yes (27)	80
GL50803_103676	Tubulin alpha chain	14037.4	9719.3	Yes (32)	—
GL50803_40817	Actin	328.2	na	Yes (40)	8
GL50803_15124	Dynein light chain roadblock	na	956.9	Yes (31)	—
GL50803_102101	Kinesin‐3	2989.0	1674.7	Yes (36)	—
GL50803_112846	Kinesin‐3	2285.6	1537.0	Yes (36)	—
Disc proteins					
GL50803_11654	Alpha‐1 giardin	6390.5	2271.4	Yes^c^ (36)	—
GL50803_7796	Alpha‐2 giardin	922.2	na	Yes^c^ (36)	—
GL50803_14551	Alpha‐6 giardin	1803.9	na	Yes^c^ (36)	—
GL50803_103373	Alpha‐7.1 giardin	3414.2	1213.0	Yes^c^ (36)	—
GL50803_114119	Alpha‐7.2 giardin	5603.4	1727.8	Yes^c^ (36)	—
GL50803_114787	Alpha‐7.3 giardin	6524.5	1718.1	Yes^c^ (36)	—
GL50803_5649	Alpha‐10 giardin	214.3	na	Yes^c^ (36)	—
GL50803_17153	Alpha‐11 giardin	3847.8	na	Yes^c^ (36)	—
GL50803_15097	Alpha‐14 giardin	4661.3	3885.9	Yes^c^ (36)	—
GL50803_4812	Beta‐giardin	31803.6	na	Yes^c^ (36)	—
GL50803_86676	Delta giardin	4101.2	na	Yes^c^ (36)	—
GL50803_17230	Gamma giardin	1413.9	na	Yes^c^ (36)	—
GL50803_4410	SALP‐1	1455.1	na	No	—
GL50803_15410	Ankyrin repeat protein 1	4638.3	na	Yes (39)	—
GL50803_16343	Median body protein	995.3	na	No	—
Cathepsin‐like proteinases					
GL50803_14019	Giardipain1	120.4	na	Yes^d^ (6)	—
GL50803_16160	Giardipain2	124.3	na	Yes^d^ (6)	—
GL50803_16779	Giardipain3	120.4	na	Yes^d^ (6)	—
Antioxidant system enzymes					
GL50803_10358	A‐type flavoprotein	550.2	1593.8	No	—
GL50803_14521	Thioredoxin‐dependent peroxiredoxin	446.2	1777.1	Yes (30)	35, 53
GL50803_15383	Thioredoxin‐dependent peroxiredoxin	661.5	na	Yes (30)	35, 53
GL50803_16076	Thioredoxin‐dependent peroxiredoxin	446.2	1777.1	Yes (30)	35, 53
GL50803_33769	NADH oxidase	1897.2	877.4	No	—
GL50803_9719	FAD/FMN dependent oxidoreductase	600.9	na	No	—
Cell cycle progression					
GL50803_17400	Cyclin	178.2	na	Yes^e^ (38)	—
GL50803_15409	Kinase, NEK	4106.6	na	Yes^e^ (38)	—
GL50803_15411	Kinase, NEK	2425.3	na	Yes^e^ (38)	—
GL50803_11390	Kinase, NEK	1142.1	na	Yes^e^ (38)	—
GL50803_14742	Kinase, NEK	162.7	na	Yes^e^ (38)	—
GL50803_26199	Kinase, NEK	158.4	na	Yes^e^ (38)	—
GL50803_113030	Kinase, NEK	142.0	na	Yes^e^ (38)	—

^a^ http://microvesicles.org/index.html

^b^not available

^c^Giardins are included within the annexin family (Einarsson et al., 2016)

^d^Considering that Giardipains are extracellular proteinases (Argüello‐García et al. 2023)

^e^NEK kinases: NIMA‐associated kinases

**FIGURE 3:**
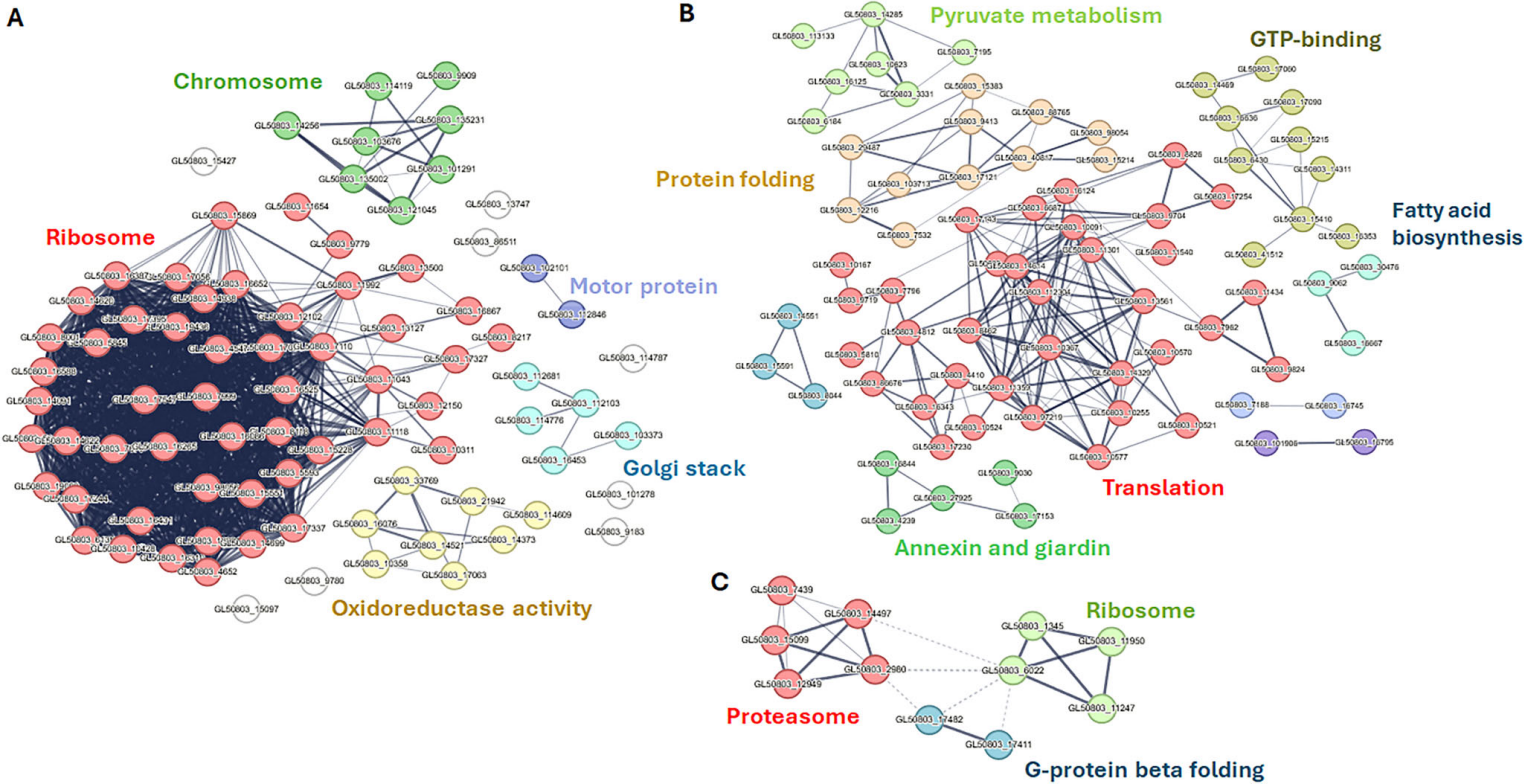
**Protein–protein interaction (PPI) network of proteins found in EVs derived from TYI‐33 and DMEM cultured trophozoites. A)** Proteins shared by EVs from both culture media, **B)** Unique proteins from TYI‐33 and **C)** DMEM.

To explore the functional profile of EV protein cargo from trophozoites cultured in TYI‐33 or DMEM, we performed Gene Ontology (GO) enrichment analysis using a false discovery rate (FDR) threshold of 1e^−07^ as the statistical criterion for valid identification (Figure 2C,D). In both EV preparations, GO analysis revealed significant enrichment of biological processes related to translation, peptide metabolism, and macromolecular biosynthesis. At the molecular function level, structural molecule activity, ribosome biogenesis, and rRNA binding were prominently represented. Regarding cellular components, ribosomal subunits, particularly large subunits, were consistently enriched in both samples. Additionally, protein–protein interaction (PPI) network analysis of the 82 proteins shared between TYI‐33 and DMEM‐derived EVs revealed strong clustering into functional categories, including ribosomal proteins (55), chromatin‐associated proteins (8, including histones H2A, H2B, H3, and H4), oxidoreductases (8), Golgi‐stacking proteins (5), and motor proteins (2) (Figure 3A).

To gain deeper insights into the biological relevance of the EV cargo, we further classified the proteomic data according to the known functional roles of the identified proteins in *G. duodenalis* biology (Table S1). This analysis revealed notable differences between the EVs derived from trophozoites cultured in TYI‐33 and those maintained in DMEM, and in comparison, with EVs reported in other organisms. For instance, the unique proteome of TYI‐33‐EVs (145 proteins) included several proteins with key biological functions: disc‐associated proteins (7), cell cycle progression (7), and cathepsin B‐like cysteine proteases (3) herein named as giardipain‐1, ‐2, and ‐3 (GL50803_14019, GL50803_16160, and GL50803_16779) none of which were detected in DMEM‐EVs. Additional TYI‐33‐exclusive proteins were associated with translation (35), pyruvate metabolism (7), protein folding (11), GTP‐binding (10), annexin‐like giardins (5), and fatty acid biosynthesis (3) (Figure 3B). In contrast, the 15 proteins uniquely identified in DMEM‐EVs were primarily linked to proteasome function (5), ribosomal activity (4), and G‐protein β‐fold folding (2) (Figure 3C).

To contextualize these findings, we searched the Vesiclepedia v5.1 database to determine whether the *Giardia* EV proteins have homologs in EVs from other organisms. We found that many of the glycolytic and arginolytic enzymes present in *Giardia* EVs such as enolase, pyruvate kinase, aldolase, and glyceraldehyde 3‐phosphate dehydrogenase are commonly found in EVs from diverse eukaryotic cells, from protists to mammals (5‐30 EV proteomes), alongside cytoskeletal elements (e.g., tubulin, actin; 27–40 EV proteomes), antioxidant enzymes (e.g., peroxiredoxins; 30 EV proteomes), and cell cycle regulators (38 EV proteomes). Interestingly, parasite‐specific proteins like giardins (in 36 EV proteomes as annexins), the median body protein, as well as the cathepsin B‐type cysteine proteases giardipains 1–3 (in 6 EV proteomes), were also identified. Altogether, 39 of the 46 proteins listed in Table 1 had homologous counterparts in EVs derived from other organisms, underscoring a conserved functional repertoire among EVs from diverse species.

**FIGURE 4:**
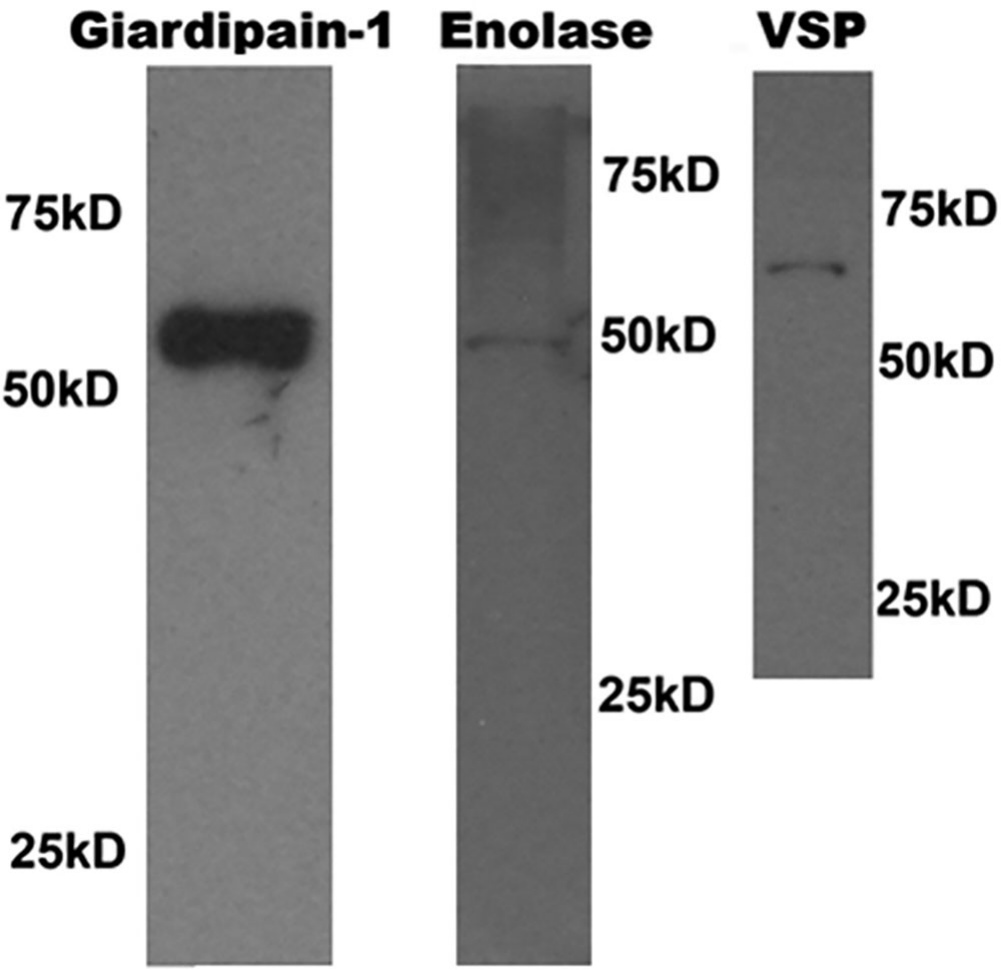
**Detection of virulence‐associated proteins in *G. duodenalis*‐derived TYI‐33‐EVs by Western blot**. Representative immunoblots showing the presence of giardipain‐1, enolase, and VSP9B10A in protein extracts from EVs isolated from *G. duodenalis* trophozoites. Specific bands corresponding to giardipain‐1 in its dimeric form (∼50 kDa), enolase (∼47 kDa), and VSP9B10A (∼65–72 kDa) were detected, confirming the selective enrichment of these virulence‐associated proteins within the *G.duodenalis* EVs cargo.

Complementing proteomics studies, Western blot assays were performed on TYI‐33‐EVs. This approach confirmed the presence of three relevant virulence factors: giardipain‐1, enolase, and variant‐specific surface protein (VSP9B10A) (Figure 4). In the case of giardipain‐1 (MW of mature form: 25 kDa), it was observed in a likely dimeric conformation with an approximate MW of 50 kDa. Also, enolase was detected as a band at 47 kDa and a smear at 75–120 kDa, suggesting the presence of the dimer. Meanwhile VSP9B10A was reactive in its typical size at 65–72 kDa. All these proteins have been consistently associated with the parasite's pathogenic mechanisms during host interaction (Argüello‐García and Ortega‐Pierres 2021). These findings support the hypothesis that *Giardia* utilizes these vesicles to deliver virulence‐associated molecules directly to the intestinal microenvironment, thereby enhancing its capacity to modulate host responses and promote epithelial damage.

### 3.3 Internalization of G. *duodenalis* EVs by IEC‐6 Cells

**FIGURE 5:**
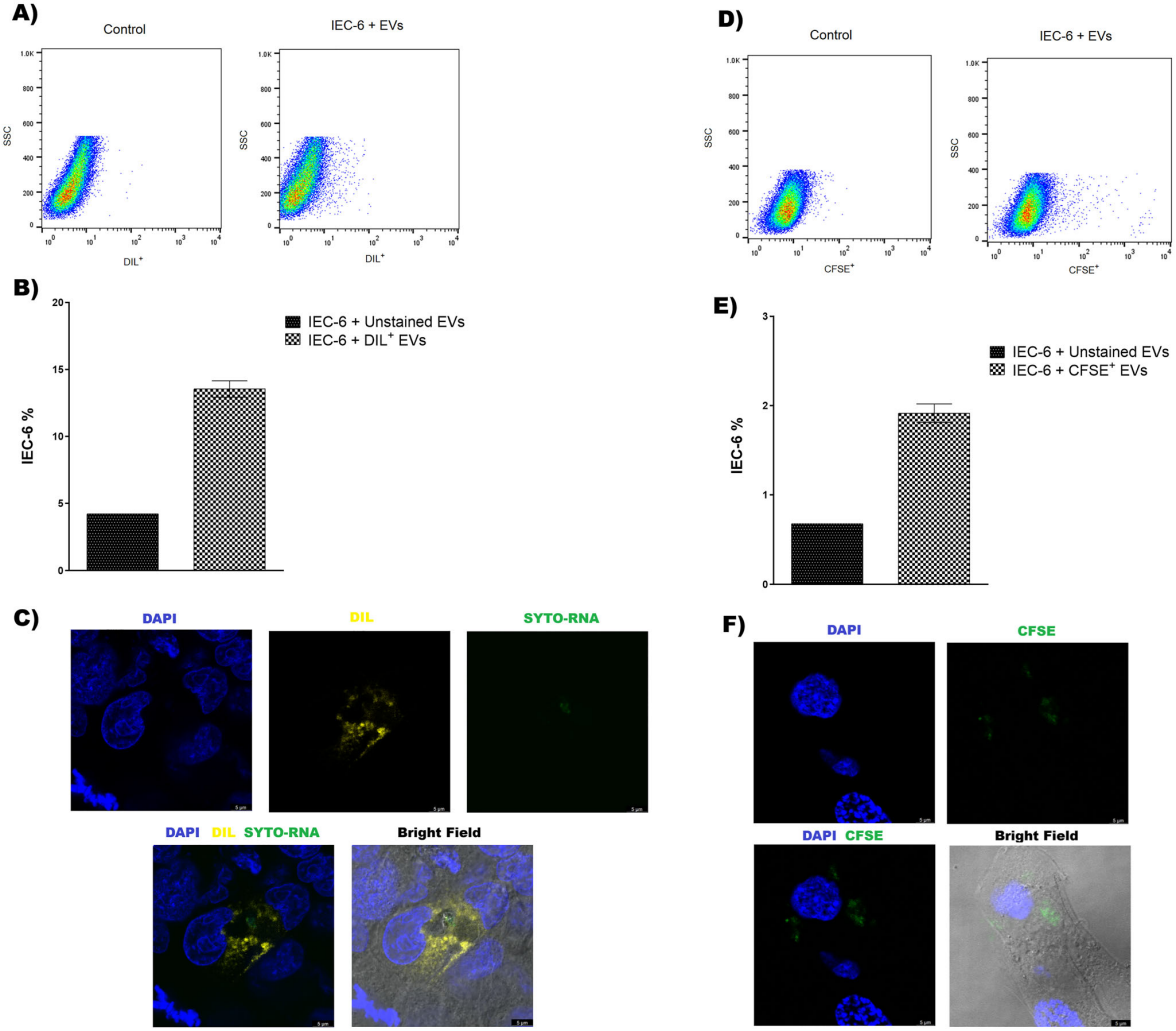
**Internalization of *G. duodenalis* TYI‐33‐EVs by intestinal epithelial cells (IEC‐6)**. **(A**–**C)** TYI‐33–derived EVs were labeled with the membrane dye DiI for flow cytometry analysis and, in parallel experiments, co‐labeled with the membrane dye DiI and the RNA‐specific dye SYTO RNA. Labeled EVs were then incubated with IEC monolayers for 3 h at 37°C. **A)** Representative flow cytometry dot plots analyses illustrating EVs uptake by IECs. **B)** Quantification of Dil‐positive IECs, expressed as the percentage of cells exhibiting fluorescence, indicating EV internalization. **C)** Confocal fluorescence microscopy images showing IECs with internalized Dil‐labeled EVs (yellow) and associated RNA cargo stained with Syto RNA (green). Nuclei were counterstained with DAPI (blue). **(D**–**F)** Protein cargo of TYI‐33‐EVs was labeled with CFSE and incubated with IEC monolayers for 3 h at 37°C. **D)** Representative flow cytometry dot plots illustrating the uptake of CFSE‐labeled EVs by IECs. **E)** Quantification of CFSE‐positive IECs, expressed as the percentage of fluorescent cells, indicating internalization of EVs. **F)** Confocal fluorescence microscopy images showing IECs with internalized CFSE‐labeled EVs (green). Nuclei were counterstained with DAPI (blue). Experiments were performed in triplicate and statistical analysis was carried out using Mann–Whitney test.

IEC‐6 cell monolayers were incubated with fluorescently labeled TYI‐33‐EVs and analyzed by flow cytometry and confocal microscopy to evaluate their internalization by intestinal epithelial cells. After a 3 h exposure to Dil‐labeled TYI‐33‐EVs, flow cytometric analysis showed that approximately 12% of IEC‐6 cells were Dil^+^, indicating uptake of EVs (Figure 5A,B). To simultaneously assess membrane incorporation and nucleic acid cargo delivery, EVs were labeled with both Dil and SytoRNA. Confocal microscopy revealed co‐localization of both fluorescent signals within the cytoplasm, confirming that internalized EVs delivered RNA‐containing cargo into host cells (Figure 5C).

In further analysis to assess protein cargo delivery, TYI‐33‐EVs were labeled with CFSE. Following a 3 h incubation with IEC‐6 monolayers, flow cytometry detected approximately 2% CFSE^+^ cells (Figure 5D,E). Despite the lower frequency of positive cells, confocal microscopy confirmed CFSE signal within the cytoplasm, corroborating the internalization and release of TYI‐33‐EV protein contents (Figure 5F).

Collectively, these findings demonstrate that *G. duodenalis*‐derived EVs are internalized by IEC‐6 cells and are capable of transferring membrane and cytoplasmic components, supporting their role as mediators of host‐pathogen communication through vesicle‐mediated molecular transfer.

### 3.4 Alterations and Tight Junction Disruption of IEC‐6 Cells Induced by G. *duodenalis* EVs

**FIGURE 6:**
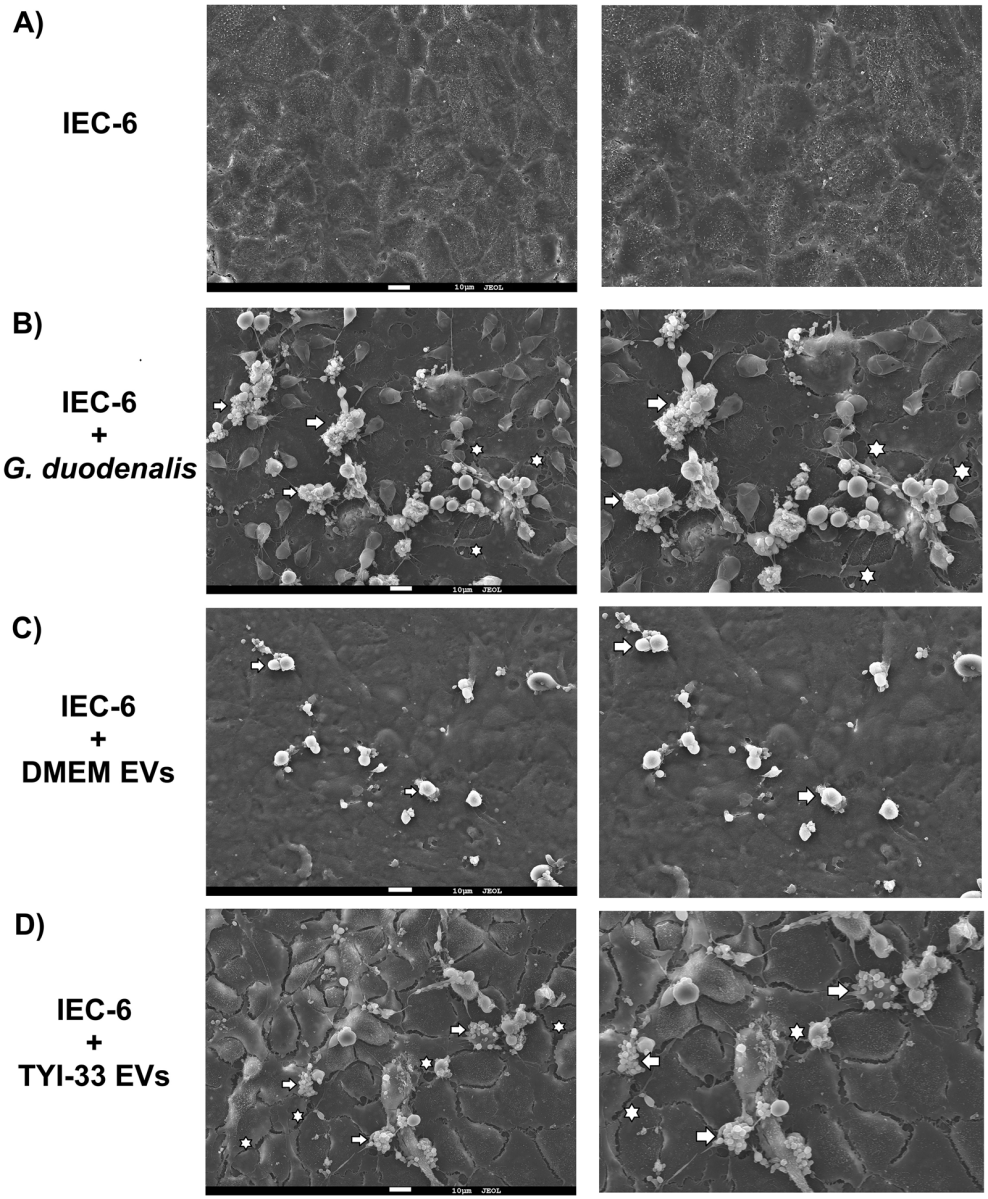
***Giardia duodenalis* EVs induce cell death in intestinal epithelial cells (IEC‐6)**. Scanning electron microscopy (SEM) images of IEC monolayers after 5 h of incubation at 37°C. **A)** Untreated control cells show an intact and confluent epithelial monolayer, with preserved morphology and junctional contacts. **B)** IECs exposed to live *Giardia* trophozoites display severe damage, including membrane blebbing and other surface irregularities. **C)** IECs incubated with 100 µg of total DMEM‐EVs protein exhibit morphological changes consistent with apoptotic processes, such as surface blebbing (arrow), membrane irregularities, and cell shrinkage. **D)** Cells treated with 100 µg of total TYI‐33‐EVs protein obtained in TYI‐33 culture exhibit pronounced structural alterations, such as intercellular separation (asterisk), blebbing, and cell shrinkage (arrow), morphological features consistent with apoptosis or stress‐induced cell death. The micrographs on the right shows a magnified view. Bar = 10 µm.

To further assess the effects of *G. duodenalis*‐derived EVs on intestinal epithelial cells, IEC‐6 monolayers were examined by scanning electron microscopy following a 5 h incubation with *G. duodenalis* EVs. Untreated IEC‐6 monolayers exhibited a continuous epithelial layer composed of closely associated cells with preserved morphology, and intact cell‐cell contacts (Figure 6A). In contrast, exposure to *G. duodenalis* trophozoites led to pronounced epithelial disruption, characterized by a widespread loss of monolayer integrity, rounding and detachment of cells, and surface blebbing (Figure 6B). Notably, epithelial alterations were also observed in monolayers exposed to parasite‐derived EVs. Cells exhibited signs of membrane blebbing after incubation with DMEM‐EVs (Figure 6C). However, in the case of TYI‐33‐EVs, the extent of epithelial disruption was as severe as the one observed with intact trophozoites, showing membrane blebbing, loss of junctional integrity, and surface vesiculation, which are commonly associated with apoptosis or severe cellular stress (Figure 6D).

**FIGURE 7:**
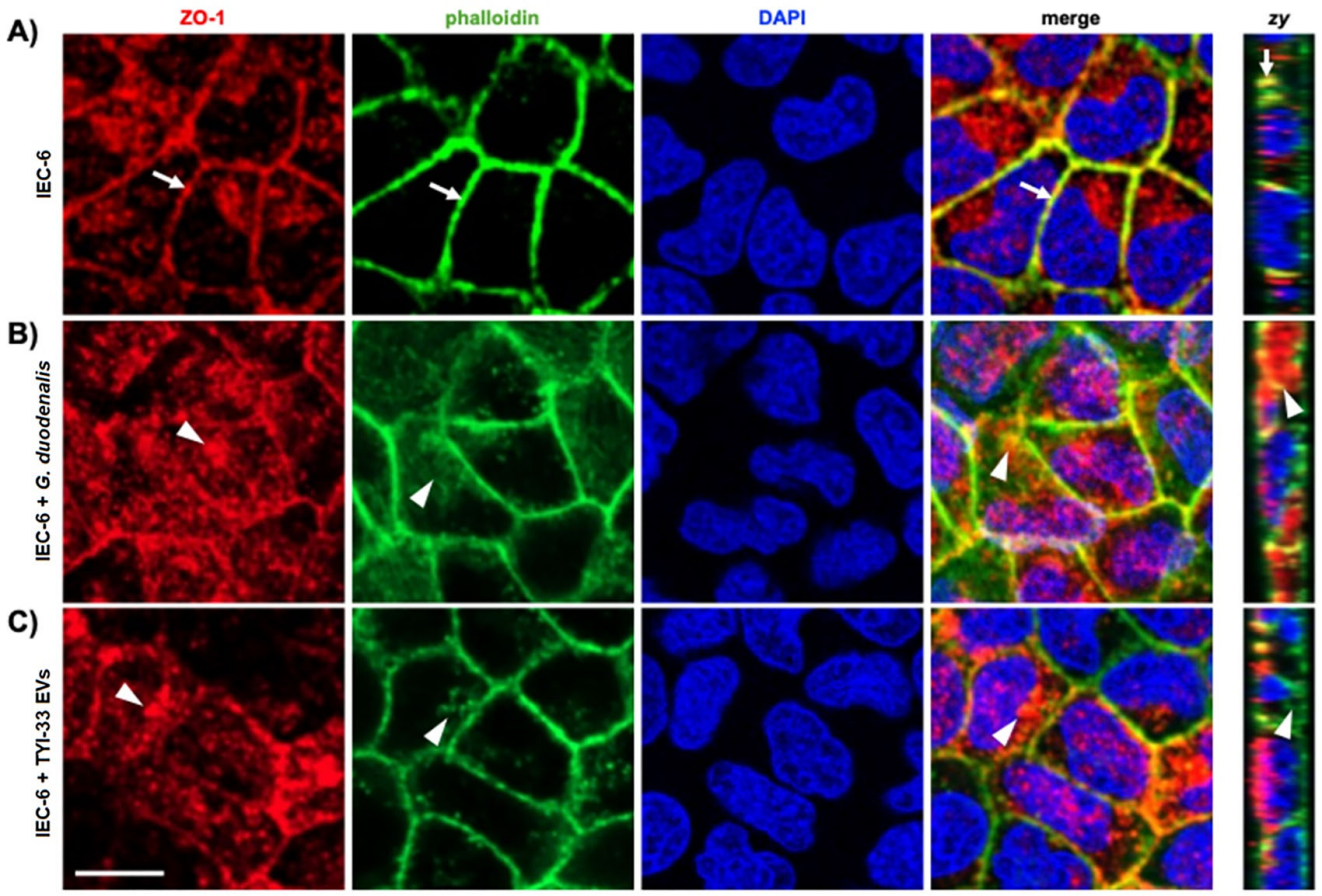
**Localization of ZO‐1 and actin in intestinal epithelial cells (IEC‐6) after incubation with *Giardia duodenalis* TYI‐33‐EVs. A)** IECs control, **B)** incubated with *G. duodenalis* live trophozoites or **C)** TYI‐33‐EVs. Preparations were analyzed with a confocal microscope considering *xy*‐ and *zy*‐planes. Arrows: ZO‐1 and polymerized actin are located at the cellular borders. Arrowheads: Incubation with either *G. duodenalis* trophozoites or TYI‐33‐EVs change their localization to the cytoplasm. Bar = 10 µm.

To determine whether EV components can disrupt the epithelial barrier, the junctional protein ‐ZO‐1 and its associated cytoskeletal protein actin were examined in IEC‐6 monolayers after a 3 h incubation with TYI‐33‐EVs by confocal microscopy. In control cells (Figure 7A), the tight junction (TJ) marker ZO‐1 and the apical actin ring were localized predominantly at the cell borders, consistent with intact TJs. However, in cells treated with live *G. duodenalis* trophozoites (Figure 7B) or TYI‐33‐EVs (Figure 7C), ZO‐1 showed a diffuse and significantly reduced signal along the plasma membrane, while polymerized actin appeared more abundant in the cytoplasm. Confocal imaging in the *zy*‐plane confirmed the loss of junctional localization, as well as the internalization of both ZO‐1 and actin (Figure 7A–C). Collectively, these changes in the distribution of junctional proteins strongly indicate that exposure to *G. duodenalis* or its EVs, particularly TYI‐33‐EVs, compromises the integrity of the epithelial barrier.

### 3.5 Intranasal Immunization With *G. duodenalis*‐derived EVs Confers Protection Against Infection in Gerbils

**FIGURE 8:**
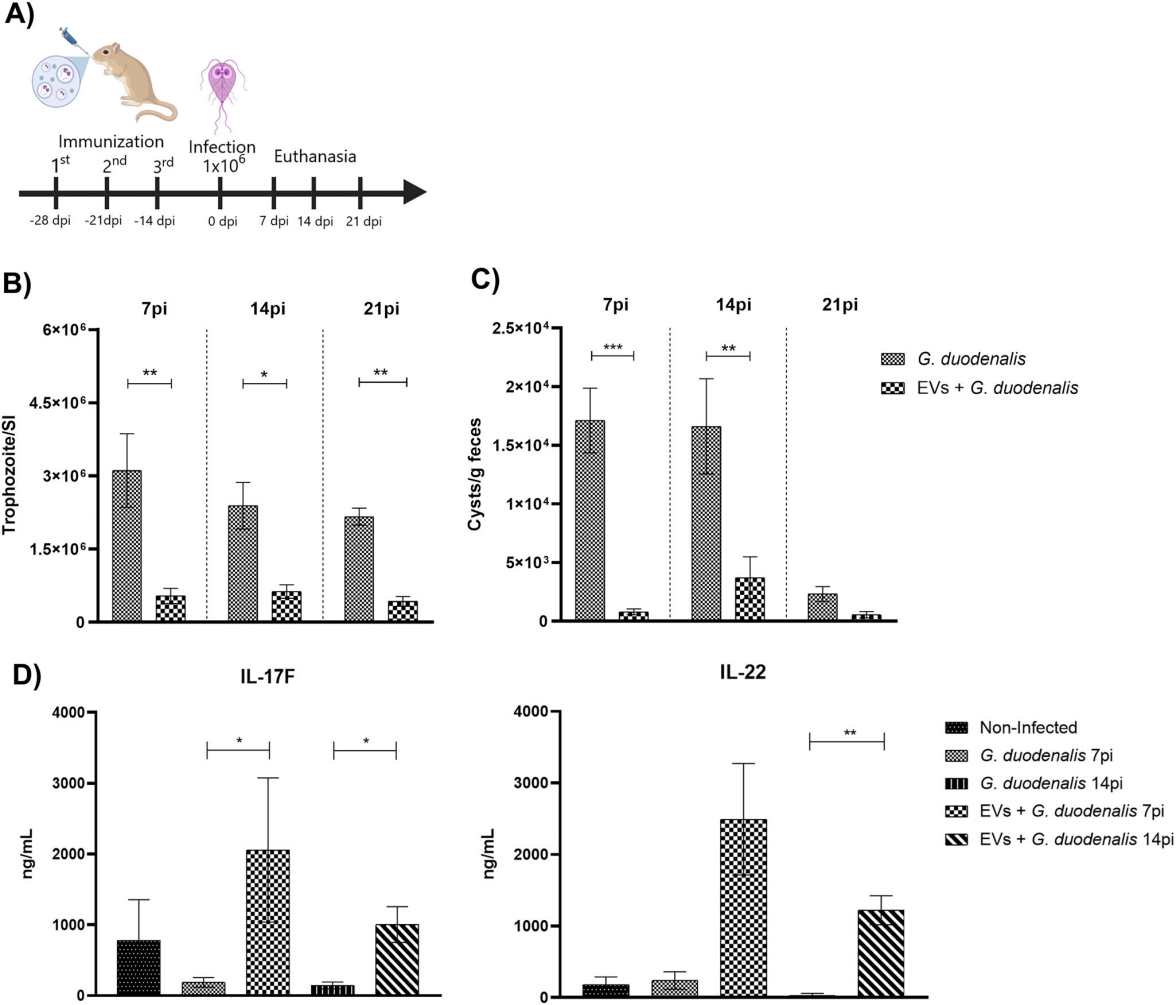
**Immunization with *Giardia duodenalis* TYI‐33‐EVs confers protection to gerbils against infection. A)** Schematic representation of the intranasal immunization protocol using 10 µg of EVs. **B)** Quantification of trophozoites recovered from the small intestine at 7, 14, and 21 dpi. **C)** Quantification of cysts recovered from fecal samples at 7, 14, and 21 dpi. **D)** Serum levels of IL‐17F and **E)** IL‐22 measured on days 7 and 14 pi. Gerbils were orally challenged with 1 × 10^6^ *G. duodenalis* trophozoites following immunization. Data are presented as mean ± SEM. Statistical significance was assessed using the **B,C)** Mann–Whitney test or **D)** Kruskal–Wallis test with Dunn's *post hoc*. *p < 0.05, **p < 0.01, ***p < 0.001. Results are representative of three independent experimental replicates. (*n* = 5 gerbils/group).

To evaluate the protective effect of *G. duodenalis* TYI‐33‐EVs, gerbils were immunized with 3 intranasal weekly doses of 10 µg total EV‐derived protein *per* dose. Two weeks after the last immunization, animals were orally challenged with live *G. duodenalis* trophozoites (Figure 8A). Parasite burden was assessed at 7‐, 14‐, and 21‐days post‐infection (dpi) by quantifying both intestinal trophozoites and faecal cyst excretion.

Remarkably, intranasal immunization with *G. duodenalis* EVs resulted in a pronounced reduction in both intestinal trophozoite load and cyst shedding at all evaluated time points when compared to non‐immunized controls. Quantitation of trophozoites from the proximal small intestine revealed that TYI‐33‐EVs‐immunized animals consistently harboured fewer parasites, suggesting that early stages of parasite colonization and/or replication were effectively controlled. Similarly, the number of cysts recovered from faeces indicated a substantial inhibition of encystation and therefore a reduction in transmission potential (Figure 8B,C).

Among the soluble immune mediators associated with this protective response, we observed a marked increase in serum IL‐17F concentrations on days 7 and 14 post‐infection, along with elevated levels of IL‐22 at day 14 pi (Figure 8D). These cytokines are known to play key roles in mucosal defence and epithelial barrier maintenance, and these results underscore their likely contribution to vaccine‐induced protection against *G. duodenalis*.

**FIGURE 9:**
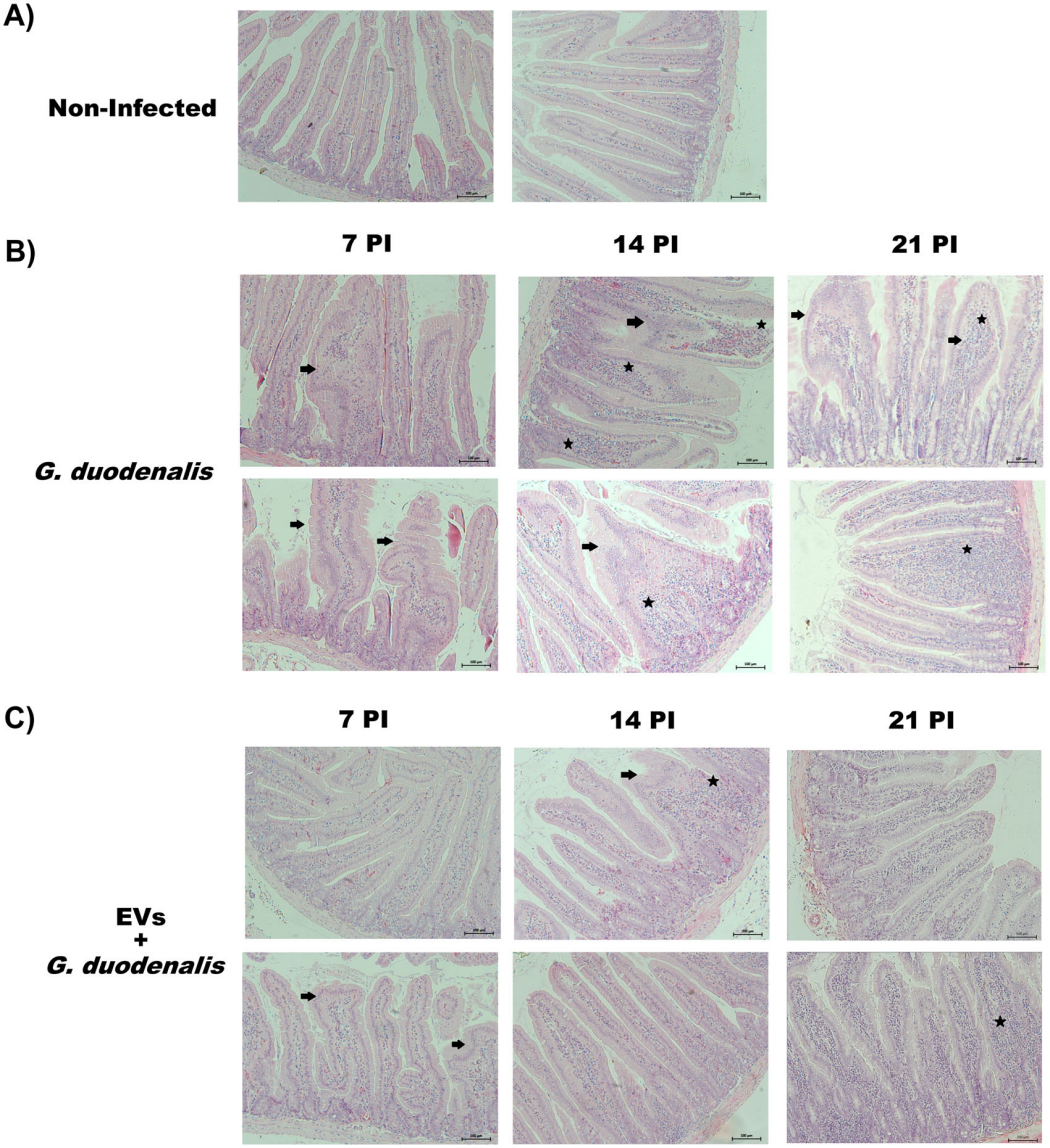
**Immunization with *Giardia duodenalis* TYI‐33‐EVs prevents histological alterations in the duodenum in *G. duodenalis* infected gerbils**. Representative H&E‐stained micrographs of duodenal tissue from gerbils at 7, 14, and 21 dpi. **A)** Duodenal sections from non‐infected animals showing normal mucosal architecture with intact villi and no pathological alterations. **B)** Duodenal sections from gerbils infected with *G. duodenalis* WB trophozoites, displaying marked histopathological changes including villus hypertrophy, shortening and fusion, crypt hyperplasia, and inflammatory cell infiltration. These alterations were most pronounced on day 14pi. **C)** Duodenal sections from TYI‐33‐EV‐immunized gerbils and infected with WB trophozoites, exhibited substantially reduced histological damage, with less frequent and less severe mucosal alterations compared to non‐immunized controls. Gerbils were orally challenged with 1 × 10^6^ *G. duodenalis* trophozoites following immunization. **(→)** Represents villus fusion; **(★)** indicates inflammatory cell infiltration. Scale bar: 100 µm. (*n* = 5 gerbils/group).

To further assess the protective effect induced by TYI‐33‐EV immunization, histopathological analyses of duodenal tissues were performed on samples collected at 7, 14, and 21 days post‐infection. *Giardia*‐infected control animals exhibited marked disruption of the intestinal mucosa, characterized by extensive villus fusion and blunting, as well as dense inflammatory infiltrates within the lamina propria (Figure 9B). These histopathological changes were most pronounced at 14 days post‐infection and are typically associated with malabsorption and local immune activation during giardiasis.

In contrast, animals immunized with TYI‐33‐EVs displayed well‐preserved mucosal architecture throughout all points examined. Intestinal villi largely maintained their normal morphology, and inflammatory cell infiltration was markedly reduced compared to that observed in non‐immunized infected animals (Figure 9C). Although occasional mild structural alterations were observed, they were considerably less severe and showed minimal immune cell accumulation. These findings suggest that EV immunization not only reduces parasite burden but also mitigates the accompanying mucosal inflammation and epithelial damage.

**FIGURE 10:**
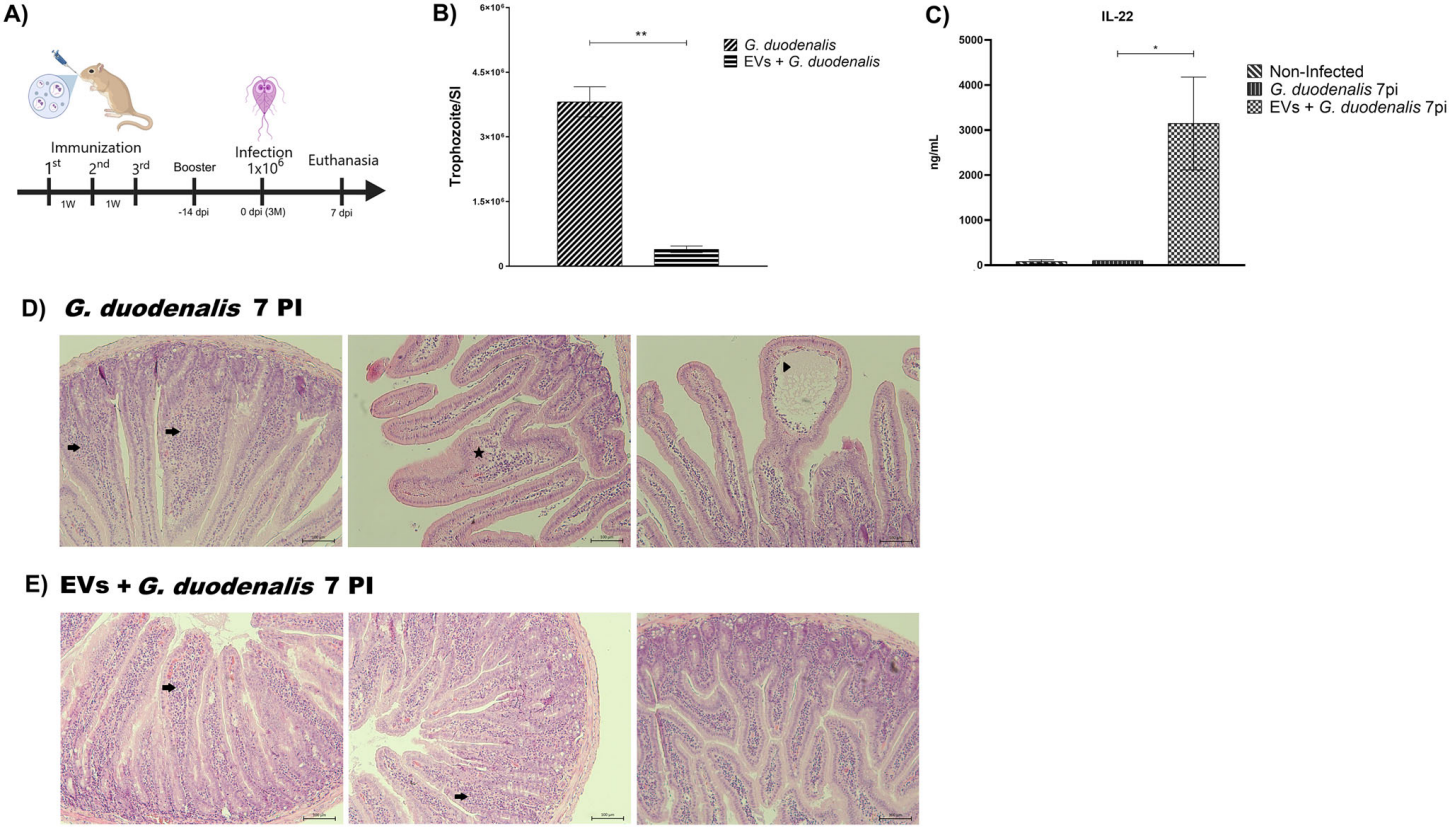
**TYI‐33‐EVs immunization induces long‐lasting immune memory against *Giardia duodenalis* infection. A)** Schematic representation of the intranasal immunization protocol with 10 µg of TYI‐33‐EVs, followed by a booster dose administered 14 days prior to infection. **B)** Quantification of trophozoites recovered from the small intestine at 7 dpi, three months after the initial immunization. **C)** IL‐22 serum levels measured 7 dpi. **D)** Duodenal sections from gerbils infected with *G. duodenalis* WB trophozoites displaying marked histopathological changes including villus hypertrophy, shortening and fusion, crypt hyperplasia, and inflammatory cell infiltration. **E)** TYI‐33‐EV‐immunized gerbils and infected with WB trophozoites, exhibited substantially reduced histological damage, with less frequent and less severe mucosal alterations compared to non‐immunized controls. Gerbils were orally challenged with 1 × 10^6^ *G. duodenalis* trophozoites. Data are presented as mean ± SEM. Statistical significance was determined using the **B)** Mann–Whitney test or **C)** Kruskal–Wallis test with Dunn's *post hoc*. **p < 0.01. **(→)** Represents villus tickening fusion; **(★)** indicates inflammatory cell infiltration; **(►)** denotes dilated lacteal vessels. Scale bar: 100 µm. (*n* = 5 gerbils/group).

To assess whether EV immunization could induce long‐term protective immunity, an additional group of gerbils was subjected to the same immunization regimen but challenged with *G. duodenalis* trophozoites three months after the last EVs immunization. To enhance the responsiveness of memory immune populations, a single booster immunization was administered 14 days prior to infection (Figure 10A). Notably, gerbils that received the booster maintained a significant reduction in intestinal parasite burden on day 14 post‐infection, similar in magnitude to that observed in the initial challenge model. This sustained protection was accompanied by elevated serum concentrations of IL‐22, highlighting the potential role of this cytokine in the induction and maintenance of protective mucosal immunity against *G. duodenalis* (Figure 10B,C). This indicates that EV‐based immunization successfully established long‐lived immune memory, capable of rapid reactivation and effectively eliminating parasite burden from TYI‐33‐EVs‐immunized gerbils. Moreover, histopathological analysis at this late time point showed that vaccinated animals exhibited reduced intestinal damage, characterized by well‐preserved villus architecture and minimal inflammatory infiltration, in contrast to the pronounced mucosal disruption observed in non‐immunized controls (Figure 10D,E). These findings underscore the potential of *Giardia* EVs to serve as immunogenic components for a mucosal vaccine that not only confers acute protection but also elicits enduring immunity and sustained tissue preservation against giardiasis.

## 4 Discussion

The present study presents compelling evidence that EVs released by *G. duodenalis* trophozoites play a pivotal role in mediating host‐pathogen interactions and represent a promising platform for the development of mucosal vaccines against giardiasis. Characterization of these EVs revealed that *G. duodenalis*‐derived vesicles exhibit a size distribution consistent with exosome‐like particles and display the classical morphology previously reported for EVs released by *Giardia* and other protozoan parasites (Twu et al. 2013; Ma'ayeh et al. 2017; Díaz‐Godínez et al. 2022; Siddiq et al. 2023). Electrophoretic and proteomic studies showed how the culture media composition influences the molecular cargo of *G. duodenalis*‐derived TYI‐33‐EVs and DMEM‐EVs. These two serum‐free media differ markedly in nutritional complexity: DMEM is a chemically defined medium containing free amino acids, vitamins, inorganic salts, glucose (dextrose), and pyruvic acid, whereas TYI‐33 (Keister 1983) is a more complex medium having trypticase (T), yeast extract (Y), an iron source (I), glucose, salts, vitamin C, and a significantly higher concentration of cysteine (0.2% versus 0.0063% cystine in DMEM). Worth of note, cystine is not processed to cysteine by trophozoites (Luján and Nash 1994) thereby *de novo* synthesis of cysteine‐containing proteins (e.g. VSPs, Giardipains) is very restricted in DMEM. In line with these differences, proteomic profiling showed that TYI‐33‐EVs contained a significantly broader and more diverse protein repertoire (227 proteins) compared to those from DMEM (97 proteins). Hence, uniquely featured proteins in TYI‐33‐EVs were associated with cytoskeletal architecture (e.g., disc components), cell cycle regulation, and notably, cathepsin B‐like cysteine proteases (giardipains‐1, ‐2, and ‐3), virulence factors which were completely absent from the DMEM EVs, a condition associated with a starvation‐like state, in contrast to TYI‐33 that mimics a nutrient‐rich intestinal environment. In other studies, relevant protistan virulence factors have been characterized within EV cargo, for instance enolase from *Trypanosoma cruzi* (Bayer‐Santos et al. 2013), serum resistance‐associated protein (SRA) of *T. brucei* (Szempruch et al. 2016), serine‐ and metallo‐proteinases of *Acanthamoeba castellani* (Gonçalves et al. 2019), Gp63 of *Leishmania donovan*i (da Silva et al. 2022) and naegleopore B and cathepsin B from *Naegleria fowleri* (Rodriguez‐Mera et al. 2024).

Previous studies examining the protein content of *G. duodenalis*‐derived EVs have reported considerable variability in the number of identified proteins, despite employing similar methodologies and culture conditions. Using LC‐MS/MS protocols, the number of detected proteins has ranged from 91 (Evans‐Osses et al. 2017), to 138 (Gavinho et al. 2020), and up to 662 (Grajeda et al. 2022), all from TYI‐33‐EVs. In the present study, we used a label‐free LC‐MS approach based on spectral pattern recognition and signal intensity quantification, which enabled the identification of 227 proteins in EVs isolated from TYI‐33–grown trophozoites, supporting the robustness and sensitivity of this analytical pipeline. Overall, the repertoire of virulence‐associated proteins identified across studies remains largely consistent, with the notable exception of variable detection of variant‐specific surface proteins (VSPs), whose presence appears to fluctuate depending on experimental conditions and possibly on strain‐specific expression profiles. However, the consistent presence of cathepsin B‐like proteases (e.g. giardipains 1–3; Argüello‐García and Ortega‐Pierres 2021) is an outstanding feature that may underlay an important part of the more pronounced cytophatic effects exerted by giardial TYI‐33‐EVs on IEC‐6 cells.

Other molecules with putative roles in pathogenesis that were found only in TYI‐33‐EVs include peptidyl‐prolyl cis‐trans isomerase B, a molecule with protein folding and maturation activities that in secreted form has been shown to induce pyroptosis in murine macrophages (Liu et al. 2021). Also included are the regulatory protein 14‐3‐3 and the glycolytic enzyme glyceraldehyde‐3‐phosphate dehydrogenase, both of which have been identified as virulent factors in exosomes from virulent strains of *Paracoccidioides brasiliensis* (Montanari Borges et al. 2024). Moreover 14‐3‐3 proteins have also been reported as conserved components in small EVs across different biological systems, where they are proposed to participate in vesicle biogenesis and signalling processes (Moyano et al. 2019). Their detection in *Giardia* TYI‐33‐EVs not only validates the vesicular origin of this component but its synthesis under nutrient competent media. Also, a serine peptidase (GL50803_15871) of unknown function in *Giardia* was uniquely present in TYI‐33 EVs; this finding is reminiscent of the proteolytic activities observed in EVs from the invasive protozoan *Naegleria fowleri*, which have been implicated in increased epithelial permeability and necrotic damage to host monolayers (Castelan‐Ramírez et al. 2025).

In addition to classical vacuolar markers such as sorting and fusing proteins, ATPases, and HSP90 present in TYI‐33‐derived EVs, several other biologically relevant proteins were identified (Table S1). Nevertheless DMEM‐derived EVs lacked most vacuolar markers with only two vesicle–fusing ATPases identified (i.e. GL50803_114776 and GL50803_112681), a fact consistent with the less complex composition and lower nutrient properties of DMEM and underscoring DMEM‐EVs as exosomes with a minimized molecular machinery. Notably, the high abundance of ribosomal proteins within the EV cargo may reflect either ribosome‐specific autophagic processes or the selective packaging of translational machinery, potentially aimed at modulating host cell protein synthesis and altering host cellular phenotypes (Gavinho et al. 2020; Hide et al. 2022). Another key molecule identified was the vesicular trafficking protein dynamin, a modular GTPase involved in membrane scission events, which has been previously implicated in the endocytosis of giardial EVs by Caco‐2 cells (Sabatke et al. 2025). Strikingly, EVs from both media contained the complete set of canonical histones (H2A, H2B, H3, and H4). The presence of these nuclear proteins in EVs suggests their active export, and if presented on the vesicle surface, they may function as microbe‐associated molecular patterns (MAMPs). Upon interaction with host cells such as enterocytes, macrophages, or dendritic cells, these histones could engage pattern recognition receptors including Toll‐like receptors (TLRs) triggering innate immune responses and promoting inflammation (Singh et al. 2025). In support of this, macrophages exposed to *G. duodenalis* EVs exhibit elevated transcription of pro‐inflammatory cytokines IL‐1β, IL‐6, and TNF‐α, via activation of the MAPK, AKT, and NF‐κB signaling pathways (Zhao et al. 2021b).

As previously noted, *G. duodenalis*‐derived EVs are internalized by intestinal epithelial cells, facilitating the delivery of both membrane‐bound and luminal cargo (Sabatke et al. 2025). Here, we used the IEC‐6 cell line, a non‐tumorigenic, small intestine‐derived epithelial model, which we consider more physiologically relevant than commonly used cancer cell lines. Given that *G. duodenalis* colonizes the upper small intestine, IEC‐6 cells offer a more appropriate in vitro system to investigate parasite‐epithelial interactions. Consistent with earlier reports (Gavinho et al. 2020; Yang et al. 2024), our observations confirmed the uptake of *Giardia* EVs by IEC‐6 cells, supporting the notion that these vesicles serve as active modulators of host cell physiology. Continuous exposure of the intestinal mucosa to these vesicles throughout infection likely results in a sustained and cumulative transfer of bioactive molecules, which can influence epithelial signaling pathways, metabolic homeostasis, and barrier integrity. Such modulation may impair absorptive capacity and contribute to hallmark features of giardiasis, including nutrient malabsorption, gastrointestinal dysfunction, and chronic inflammation.

Further supporting their pathogenic potential, TYI‐33‐derived EVs were found to compromise epithelial integrity. Immunofluorescence analysis of IEC monolayers exposed to these vesicles revealed a marked disruption of TJ architecture, evidenced by the redistribution of the TJ marker ZO‐1 and the actin ring. While control cells displayed ZO‐1 and actin localized sharply at intercellular borders, EV‐treated monolayers exhibited a diffuse cytoplasmic staining pattern, consistent with junctional disassembly and impaired barrier function. These findings are consistent with similar EV‐mediated effects reported for other pathogens, for example, *Trichinella spiralis* and *Plasmodium* EVs have been shown to disrupt TJs in the intestinal and blood‐brain barriers, respectively, whereas EVs from *Bacteroides fragilis* primarily affect adherent junctions in the intestinal epithelium (Mantel et al. 2016; Zakharzhevskaya et al. 2017; Wang et al. 2022; Pais and Penha‐Gonçalves 2023). Together, this evidence highlights a conserved strategy among diverse pathogens whereby their EVs disrupt intercellular junctions compromising host barrier integrity and promoting pathogenesis.

The disruption of epithelial monolayers incubated with EVs was corroborated by SEM analyses, which revealed pronounced morphological alterations, including membrane blebbing, fragmentation of the epithelial monolayer, and complete loss of cell‐cell junctions. Importantly, these changes occurred in the absence of direct trophozoite contact, indicating that EVs alone are sufficient to induce epithelial injury. Notably, these structural disruptions may be mechanistically linked to the presence of *Giardia* virulence factors which include giardipain‐1 a cathepsin B‐like cysteine protease, and enolase, a moonlighting protein previously associated with host‐parasite interactions (Franco‐Serrano et al. 2021; Barroeta‐Echegaray et al. 2022; Quezada‐Lázaro et al. 2022). As these molecules have been previously associated with epithelial damage, their delivery *via* EVs offers a plausible explanation for the alterations observed in IEC monolayers (Ortega‐Pierres et al. 2018; Barroeta‐Echegaray et al. 2022). A similar mechanism has also been reported in *Trichomonas vaginalis*, where the metalloprotease GP63 plays a critical role in host colonization. This protease is likewise released via EVs and delivered to the urogenital epithelium, suggesting that the EV‐mediated transport of proteolytic effectors is an important component of the parasite's capacity to interact with and modify mucosal surfaces (Ma et al. 2011; Nievas et al. 2018). In the case of giardiasis, the capacity of EVs to deliver pathogenic effectors and induce epithelial disruption suggests a central role in early colonization, epithelial dysfunction, and the symptomatic outcomes of *Giardia* infection.

Additionally, the differences observed in IEC‐6 monolayers treated with TYI‐33‐EVs compared to those exposed to DMEM‐EVs further highlight this effect. Our findings indicate that environmental conditions not only influence EV composition but also their biological activity. The structural alterations observed in the epithelial cells upon interaction with *Giardia* EVs closely resembled those induced by contact of trophozoites with IEC‐6 epithelial cell monolayers, supporting the notion that *Giardia* EVs can independently mediate epithelial injury. This in turn confirms that EVs carry bioactive molecules capable of disrupting epithelial homeostasis regardless of parasite presence.

Besides the role of *Giardia*‐derived EVs in parasite virulence due to their capacity to deliver proteolytic effectors that disrupt epithelial integrity and modulate host responses, the promising results to use them as an immunological tool was shown in this study. The complex and native antigenic cargo carried by EVs, coupled with their particulate structure, enables their recognition and uptake by APCs, shaping host immunity. This dual nature of *Giardia*‐derived EVs, functioning both as mediators of pathogenicity and potential immunogenic platforms, has led to growing interest in their evaluation as vaccine candidates.

In this study, we investigated the capacity of *Giardia*‐derived EVs to induce protection against giardiasis. Intranasal immunization with EVs conferred robust protection in the gerbil model. Although this strategy did not result in complete parasite clearance or accelerated elimination by the host, it led to sustained and significant reductions in both intestinal trophozoite burdens and fecal cyst shedding throughout the course of infection. These findings are consistent with prior studies using recombinant antigens from other organisms (Davids et al. 2019; Ihara et al. 2022; Ihara et al. 2024), which have likewise demonstrated protective efficacy when administered via mucosal routes.

Accordingly, IL‐17F and IL‐22 are key cytokines involved in maintaining intestinal mucosal homeostasis and host defence against extracellular pathogens. IL‐17F, mainly produced by Th17 cells and ILC3s, promotes epithelial secretion of antimicrobial peptides and chemokines, enhancing neutrophil recruitment and mucosal protection (Keir et al. 2020; Mills 2023). In parallel, IL‐22, produced by Th17, Th22, and ILC3s, acts directly on epithelial cells to induce proliferation, tissue repair, and antimicrobial responses, thereby strengthening barrier integrity (Keir et al. 2020). In the context of *G. duodenalis* infection, the induction of IL‐17 signaling has been identified as a critical component of parasite clearance, as demonstrated in both human and murine models (Dann et al. 2015; Dreesen et al. 2014; Saghaug et al. 2015; Paerewijck et al. 2017 Paerewijck et al. 2019). This cytokine axis bridges innate and adaptive immune responses, particularly by supporting Th17‐driven IgA production, which is essential for parasite elimination. Although the role of IL‐22 in giardiasis is less well characterized, its established functions in promoting mucosal resilience and restricting pathogen dissemination render it a plausible effector involved in vaccine‐mediated protection.

In the present study, the concurrent upregulation of IL‐17F and IL‐22 in TYI‐33‐EV‐immunized animals supports their involvement in mounting a protective mucosal immune response. Specifically, IL‐17F levels increased as early as day 7 post‐infection and remained elevated through day 14, a period during which a significant reduction in parasite burden was observed. In contrast, IL‐22 levels peaked at day 14, potentially reflecting its role in epithelial restitution and barrier repair following infection‐induced injury. Nonetheless, a more precise understanding of the spatial and temporal dynamics of these cytokines would benefit from in situ detection approaches, which could elucidate the specific cellular sources and tissue distribution of IL‐17F and IL‐22, ultimately clarifying their contributions to the immune protective mechanisms elicited by *Giardia*‐derived EVs.

Histopathological analysis revealed preserved duodenal architecture and reduced mucosal inflammation in immunized animals, indicating that TYI‐33‐EVs not only prevent colonization but also mitigate tissue damage. These findings are consistent with previous mucosal vaccination efforts using recombinant *Giardia* antigens that have shown to induce protection against giardiasis, targeting antigens expressed during either the trophozoite or cyst stages of *G. duodenalis*. These include recombinant proteins such as α1‐giardin, variant‐specific surface proteins (VSPs), enolase, and cyst wall protein 2 (Jenikova et al. 2011; Radunovic et al. 2017; Serradell et al. 2018; Serradell et al. 2019; Ihara et al. 2022), which have demonstrated protective efficacy and modulation of local immune responses. Importantly, many of these same antigens such as α1‐giardin, VSPs, giardipain‐1 and enolase were identified in our proteomic analysis of TYI‐33‐EVs, suggesting that the vesicles incorporate immunodominant proteins that can elicit protective immunity. Indeed, proteases such as cathepsin B have been investigated as vaccine candidates in other parasitic models (e.g., *Schistosoma mansoni*, *Fasciola hepatica)*, highlighting the importance of identifying giardipain‐1 within the EV composition (Ricciardi et al. 2016; Wesołowska et al. 2018).

In this scenario, *Giardia*‐derived EVs are promising candidates for mucosal vaccination, as they package a wide variety of parasite‐derived molecules that reflect the parasite's antigenic landscape. Their continuous release throughout infection may support prolonged immune stimulation and the development of memory responses. By presenting multiple parasite‐derived immunogens in their natural conformation, *Giardia* EVs offer a multifaceted immunological stimulus that may outperform single recombinant antigens in eliciting robust mucosal protection. Given that *G. duodenalis* is a parasite that colonizes the intestinal mucosa, protection at this anatomical site is of critical importance. Thus mucosal immunization routes, particularly oral and intranasal, have been prioritized, as they allow for the induction of local immune responses at the site of parasite infection (Larocque et al. 2003; Abdul‐Wahid and Faubert. 2007; Serradell et al. 2018; Davids et al. 2019; Serradell et al. 2019; Ihara et al. 2022; Zhou et al. 2022; Ihara et al. 2024).

Intranasal vaccination offers multiple practical advantages, particularly for enteric and mucosa‐associated pathogens. By avoiding antigen exposure to the extreme pH and enzymatic degradation of the gastrointestinal tract, it allows for lower antigen doses while still inducing robust local immune responses. Moreover, this route not only promotes the generation of secretory IgA but also supports the differentiation and long‐term maintenance of tissue‐resident memory T cells within mucosal sites, providing durable protection and facilitating rapid recall responses upon reinfection (Lycke 2012; Jabbal‐Gill 2010; Lavelle and Ward 2022). Within this framework, the finding that intranasal delivery of *Giardia*‐derived EVs confers intestinal protection underscores the relevance of targeting the respiratory mucosa as a strategy to elicit systemic and mucosal immunity, with broad implications for controlling *Giardia* and induce other enteric immune responses (Ferreirinha et al. 2014: Ferreirinha et al. 2016; Hwang et al. 2018 Zhang et al. 2020; Tsujii et al. 2024).

EVs are inherently non‐replicative, and carry a complex repertoire of immunogenic molecules, making them suitable for mucosal delivery and allowing them to overcome several limitations of live‐attenuated or recombinant subunit vaccines. In addition, EVs portray complex antigenic mosaics that are continuously released throughout the course of infection, providing a dynamic and physiologically relevant source of parasite‐derived antigens. This property has led to their exploration as novel vaccine platforms against both bacterial and parasitic infections (Beauvillain et al. 2009; Choi et al. 2015; Maia et al. 2021; Martin‐Jaular et al. 2011). Indeed, outer membrane vesicles (OMVs) derived from *Neisseria meningitidis* are already licensed for human use, providing proof of concept for the clinical applicability of vesicle‐based vaccines (Micoli et al. 2024; Velimirov and Velimirov. 2024)

One of the primary goals of vaccination is the induction of long‐lasting immune memory. In our delayed challenge model using gerbils, EV‐immunization demonstrated protective efficacy, with vaccinated animals successfully controlling infection several months post‐immunization, evidencing functional memory immune responses. This sustained immunity likely results from the capacity of EVs to induce long‐lived memory T cells, alongside a possible induction of a protective Th17 immune profile. Notably, EVs derived from pathogens such as *Actinobacillus pleuropneumoniae* where they have been specifically evaluated as vaccine candidates and *Paracoccidioides brasiliensis* have been shown to induce a Th17‐skewed immune response (Montanari Borges et al. 2024; Hyun Park et al. 2025). Given the central role of Th17 immunity in protection against *Giardia* infections (Dann et al. 2015; Saghaug et al. 2015; Yordanova et al. 2019), it is reasonable to infer that EV‐based vaccination could similarly promote this immunological profile.

## 5 Conclusions

This study provides compelling evidence that *G. duodenalis*‐derived EVs function as integral mediators in host‐parasite interactions, simultaneously contributing to disease pathology and representing promising immune‐based strategies for controlling giardiasis. These vesicles can disrupt epithelial barrier integrity, which may promote the translocation of antigens derived from the microbiota and dietary sources, thereby exacerbating local inflammation and contributing to mucosal dysfunction.

The detection of virulence‐associated proteins such as giardipain‐1, enolase, and other factors within the EV cargo supports the notion that these vesicles induce host epithelial injury independently of direct trophozoite contact. Their continuous release during infection underscores their role in shaping the intestinal microenvironment, influencing both host cellular responses and microbial dynamics.

Importantly, *G. duodenalis*‐derived EVs elicited robust mucosal protection, as demonstrated by significant reductions in parasite colonization, cyst shedding, and histopathological damage. The presence of immunodominant antigens, many of which overlap with previously validated vaccine candidates, highlights the intrinsic potential of EVs to serve as antigenic platforms capable of priming host immune responses and triggering protective mucosal immunity. Long‐term protection observed in delayed challenge models further suggests the induction of durable memory responses, reinforcing the utility of EVs as vaccine delivery vehicles.

Taken together, our findings redefine *Giardia*‐derived EVs not only as effectors of pathogenesis but also as biologically relevant platforms for mucosal vaccination. Their dual capacity to modulate host‐pathogen interactions and shape protective immune responses pose them as promising candidates for the development of next‐generation vaccines against giardiasis and potentially other mucosal parasitic diseases.

## Author Contributions

G.C.R., R.A.G., and G.O.P. conceived and designed the study. Data curation was performed by G.C.R., R.A.G., E.P.S., and A.S.O. Formal analysis was conducted by G.C.R., R.A.G., E.P.S., and G.O.P. Funding acquisition was secured by G.O.P. Investigation was carried out by G.C.R., R.A.G., D.O.L., and G.O.P. Methodology was developed and optimized by G.C.R., R.A.G., D.O.L., L.S.V., D.H.C., and A.S.O. Project administration and resources were provided by G.O.P., who also supervised the work. Validation was performed by G.C.R., R.A.G., D.O.L., A.B., L.S.V., D.H.C., A.S.O., E.P.S., M.E.C., and G.O.P. The original draft was written by G.C.R., R.A.G., and G.O.P. All authors contributed to the review and editing of the manuscript and approved the final submitted version.

## Declaration of Generative AI and AI‐Assisted Technologies in the Manuscript Preparation Process

During the preparation of this work, the authors used OpenAI to assist with the translation and to improve the clarity and readability of the manuscript. After using this tool, the authors thoroughly reviewed, verified, and edited all generated content.

## Funding

This research was funded by the Secretaría de Ciencia, Humanidades, Tecnología e Innovación (SECIHTI), Mexico, under grant number CBF‐2025‐I‐3856.

## Conflicts of Interest

The authors declare no conflict of interest.

## Supporting information




**Supplementary Figure 1**: Workflow for the proteomic characterization of extracellular vesicles from *Giardia duodenalis*. **Supplementary Table 1**. Proteins of *Giardia duodenalis* EVs released by trophozoites cultured in TYI33 and DMEM media, or both, identified by LC‐MS.

## Data Availability

The data that support the findings of this study are available from the corresponding author upon reasonable request.
