# Supplementary material for: Extracellular Vesicles of Giardia duodenalis: Unravelling Their Virulence Factors and Potential to Induce Protection Against Experimental Giardiasis
Source: J Extracell Biol. 2026 Jun 11;5(6):e70155. doi: 10.1002/jex2.70155 (PMC13257884; doi:10.1002/jex2.70155)
Supplement: Supplementary file 1 — Supplementary Figure 1: Workflow for the proteomic characterization of extracellular vesicles from Giardia duodenalis. Supplementary Table 1. Proteins of Giardia duodenalis EVs released by trophozoites cultured in TYI33 and DMEM media, or both, identified by LC‐MS. [file JEX2-5-e70155-s001.docx]

**
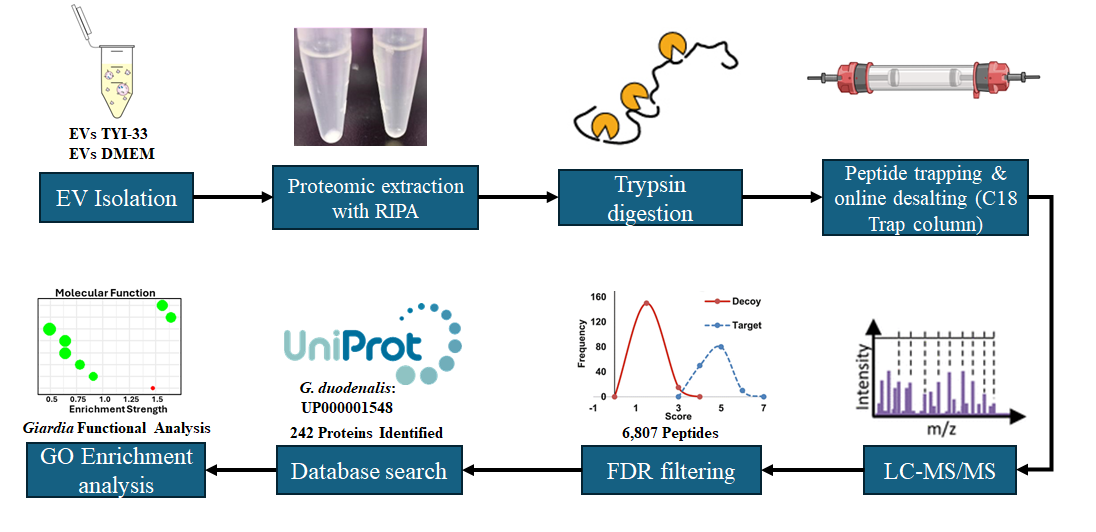
**

**Supplementary Figure 1.** Workflow for the proteomic characterization of extracellular vesicles from *Giardia duodenalis*

**Supplementary Table 1.** Proteins of *Giardia duodenalis* EVs released by trophozoites cultured in TYI33 and DMEM media, or both, identified by LC-MS.

| **Shared** | | **Unique**  **TYI33-EVs** | | **Unique**  **DMEM-EVs** | |
| --- | --- | --- | --- | --- | --- |
| **ORF** | **Protein names** | **ORF** | **Protein names** | **ORF** | **Protein names** |
| GL50803_17327 | Xaa-Pro dipeptidase | GL50803_4812 | Beta-giardin | GL50803_6022 | Ribosomal protein S20 |
| GL50803_16086 | Ribosomal protein L8 | GL50803_137716 | Axoneme-associated protein GASP-180 | GL50803_12949 | Proteasome subunit beta |
| GL50803_103373 | Alpha-7.1 giardin | GL50803_61565 | Axoneme-associated protein GASP-180 | GL50803_17411 | T-complex protein 1 subunit gamma |
| GL50803_16387 | 60S ribosomal protein L18a | GL50803_13584 | Uncharacterized protein | GL50803_14309 | Uncharacterized protein |
| GL50803_7999 | 40S ribosomal protein S3 | GL50803_17121 | Bip | GL50803_14497 | 20S proteasome alpha subunit 3 |
| GL50803_15551 | Ribosomal protein S18 | GL50803_15410 | Ankyrin repeat protein 1 | GL50803_11950 | Ribosomal protein L18 |
| GL50803_16652 | Ribosomal protein S13 | GL50803_60211 | Ribosomal protein S19e | GL50803_11247 | Ribosomal protein L13a |
| GL50803_33769 | NADH oxidase | GL50803_15409 | Kinase, NEK | GL50803_17482 | T-complex protein 1 subunit delta |
| GL50803_16453 | Carbamate kinase | GL50803_86676 | Delta giardin | GL50803_2980 | 20S proteasome alpha subunit 5 |
| GL50803_15869 | GTP-binding nuclear protein | GL50803_16745 | Axoneme-associated protein GASP-180 | GL50803_1345 | Ribosomal protein L10a |
| GL50803_11043 | Fructose-bisphosphate aldolase (Glfba) (glFBPA) (EC 4.1.2.13) (Fructose-1,6-bisphosphate aldolase) | GL50803_17153 | Alpha-11 giardin | GL50803_7439 | Ser/Thr phosphatase 2A regulatory subunit A |
| GL50803_17056 | Ribosomal protein L6 | GL50803_27925 | Ankyrin repeat protein 1 | GL50803_15099 | Proteasome subunit alpha type |
| GL50803_101278 | Uncharacterized protein | GL50803_9413 | protein disulfide-isomerase (EC 5.3.4.1) | GL50803_2101 | Cytidine deaminase (EC 3.5.4.5) (Cytidine aminohydrolase) |
| GL50803_10358 | A-type flavoprotein | GL50803_15411 | Kinase, NEK | GL50803_15124 | Dynein light chain roadblock |
| GL50803_13500 | CCT-theta | GL50803_6430 | 14-3-3 protein (GI-14-3-3) (g14-3-3) | GL50803_8903 | Copine I |
| GL50803_9779 | Uridine phosphorylase 1 | GL50803_4239 | Uncharacterized protein |  |  |
| GL50803_13127 | Proteasome subunit beta | GL50803_17090 | Trophozoite antigen GTA-1 |  |  |
| GL50803_12150 | Alanine aminotransferase | GL50803_14859 | Ankyrin repeat protein 1 |  |  |
| GL50803_11992 | T-complex protein 1 subunit epsilon (CCT-epsilon) | GL50803_9030 | Ankyrin repeat protein 1 |  |  |
| GL50803_135231 | Histone H3 | GL50803_10091 | Ribosomal protein L23 |  |  |
| GL50803_112681 | Vesicle-fusing ATPase (EC 3.6.4.6) | GL50803_88765 | Cytosolic heat shock protein 70 |  |  |
| GL50803_114776 | Vesicle-fusing ATPase (EC 3.6.4.6) | GL50803_14551 | Alpha-6 giardin |  |  |
| GL50803_16588 | Ribosomal protein P2 | GL50803_93358 | Aldehyde-alcohol dehydrogenase |  |  |
| GL50803_17547 | Large ribosomal subunit protein uL4 (60S ribosomal protein L4) | GL50803_103713 | Protein disulfide isomerase PDI4 |  |  |
| GL50803_103676 | Tubulin alpha chain | GL50803_4410 | SALP-1 |  |  |
| GL50803_17337 | Ribosomal protein P1B | GL50803_5810 | Pyridoxamine 5'-phosphate oxidase |  |  |
| GL50803_101291 | Tubulin beta chain | GL50803_10808 | Uncharacterized protein |  |  |
| GL50803_16265 | Small ribosomal subunit protein eS1 | GL50803_17230 | Gamma giardin |  |  |
| GL50803_14373 | Dynamin | GL50803_10367 | Ribosomal protein S24 |  |  |
| GL50803_14620 | 40S ribosomal protein S6 | GL50803_5795 | Leucine-rich repeat protein 1 virus receptor protein |  |  |
| GL50803_15228 | Ribosomal protein S15A | GL50803_14329 | 40S ribosomal protein S7 |  |  |
| GL50803_5845 | 40S ribosomal protein S8 | GL50803_29487 | Protein disulfide isomerase |  |  |
| GL50803_8118 | Small ribosomal subunit protein uS5 (40S ribosomal protein S2) | GL50803_11390 | Kinase, NEK |  |  |
| GL50803_114787 | Alpha-7.3 giardin | GL50803_12139 | Ankyrin repeat protein 3 |  |  |
| GL50803_14938 | Ribosomal protein L12 | GL50803_94463 | Uncharacterized protein |  |  |
| GL50803_11654 | Alpha-1 giardin | GL50803_17060 | Ankyrin repeat protein 1 |  |  |
| GL50803_10311 | ornithine carbamoyltransferase (EC 2.1.3.3) | GL50803_16343 | Median body protein (MBP) (Disc-associated protein 16343) (DAP16343) (MB protein) |  |  |
| GL50803_16310 | Ribosomal protein L27a | GL50803_7444 | UvrB/uvrC motif-containing protein |  |  |
| GL50803_114119 | Alpha-7.2 giardin | GL50803_13561 | Translation elongation factor 1-beta |  |  |
| GL50803_19436 | Ribosomal protein L7 | GL50803_7796 | Alpha-2 giardin |  |  |
| GL50803_135002 | Histone H4 | GL50803_93548 | Phospholipase B-like (EC 3.1.1.-) |  |  |
| GL50803_5947 | Ribosomal protein L35a | GL50803_41212 | Ankyrin repeat protein 1 |  |  |
| GL50803_16525 | Ribosomal protein L3 | GL50803_7843 | Uncharacterized protein |  |  |
| GL50803_15097 | Alpha-14 giardin | GL50803_10521 | arginine--tRNA ligase (EC 6.1.1.19) (Arginyl-tRNA synthetase) |  |  |
| GL50803_16431 | Ribosomal protein L19 | GL50803_15832 | Aminoacyl-histidine dipeptidase |  |  |
| GL50803_7110 | Ubiquitin | GL50803_17163 | Peptidyl-prolyl cis-trans isomerase (PPIase) (EC 5.2.1.8) |  |  |
| GL50803_17395 | Ribosomal protein L5 | GL50803_11129 | Uncharacterized protein |  |  |
| GL50803_121045 | Histone H2B | GL50803_15383 | Peroxiredoxin 1 |  |  |
| GL50803_15427 | Trophozoite antigen GTA-2 | GL50803_10570 | peptidylprolyl isomerase (EC 5.2.1.8) |  |  |
| GL50803_12102 | Elongation factor 1-gamma | GL50803_14614 | Eukaryotic translation initiation factor 5A (eIF-5A) |  |  |
| GL50803_9183 | Uncharacterized protein | GL50803_16532 | Ankyrin repeat protein 1 |  |  |
| GL50803_17244 | 60S ribosomal protein L7a | GL50803_14469 | R-SNARE 3 |  |  |
| GL50803_8001 | Ribosomal protein L15 | GL50803_9719 | FAD/FMN dependent oxidoreductase |  |  |
| GL50803_102101 | Kinesin-3 | GL50803_17249 | Uncharacterized protein |  |  |
| GL50803_14256 | Histone H2A | GL50803_15215 | Serine/threonine-protein phosphatase (EC 3.1.3.16) |  |  |
| GL50803_12981 | Ribosomal protein S5 | GL50803_8462 | Ribosomal protein L27 |  |  |
| GL50803_11118 | phosphopyruvate hydratase (EC 4.2.1.11) | GL50803_16844 | Uncharacterized protein |  |  |
| GL50803_9909 | Pyruvate, phosphate dikinase (EC 2.7.9.1) | GL50803_9861 | Uncharacterized protein |  |  |
| GL50803_112103 | Arginine deiminase | GL50803_11434 | 20S proteasome alpha subunit 2 |  |  |
| GL50803_6135 | Ribosomal protein S17 | GL50803_7962 | 20S proteasome alpha subunit 6 |  |  |
| GL50803_112846 | Kinesin-3 | GL50803_16507 | Uncharacterized protein |  |  |
| GL50803_10428 | Ribosomal protein L10 | GL50803_16795 | Vacuolar protein sorting 4b |  |  |
| GL50803_5593 | Ribosomal protein L11 | GL50803_15214 | Serine/threonine-protein phosphatase (EC 3.1.3.16) |  |  |
| GL50803_16867 | AAA family ATPase | GL50803_8826 | Glucokinase |  |  |
| GL50803_19003 | Ribosomal protein L24A | GL50803_17551 | Ankyrin repeat protein 1 |  |  |
| GL50803_114609 | pyruvate dehydrogenase (NADP(+)) (EC 1.2.1.51) (Pyruvate:NADP(+) oxidoreductase) | GL50803_101906 | Vacuolar protein sorting 4a |  |  |
| GL50803_14091 | Ribosomal protein L14 | GL50803_16353 | Uncharacterized protein |  |  |
| GL50803_17063 | Pyruvate-flavodoxin oxidoreductase | GL50803_101326 | Uncharacterized protein |  |  |
| GL50803_14699 | Ribosomal protein S23 | GL50803_16636 | Rab2b |  |  |
| GL50803_86511 | Acyl-CoA synthetase | GL50803_21628 | Uncharacterized protein |  |  |
| GL50803_98056 | Ribosomal protein L17 | GL50803_10885 | Amylo-alpha-1,6-glucosidase |  |  |
| GL50803_17054 | 60S acidic ribosomal protein P0 | GL50803_15499 | Uncharacterized protein |  |  |
| GL50803_7870 | Ribosomal protein L23A | GL50803_115478 | Ankyrin repeat protein 1 |  |  |
| GL50803_21942 | Glutamate dehydrogenase | GL50803_119672 | Uncharacterized protein |  |  |
| GL50803_14622 | 60S ribosomal protein L13 | GL50803_10577 | Nucleolar protein 56 |  |  |
| GL50803_4547 | Ribosomal protein S9 | GL50803_11359 | 40S ribosomal protein S4 |  |  |
| GL50803_16076 | Peroxiredoxin 1 | GL50803_11540 | Hexose transporter |  |  |
| GL50803_14521 | Peroxiredoxin 1 | GL50803_98054 | Heat shock protein 90 |  |  |
| GL50803_13747 | C4 group specific protein | GL50803_5375 | non-specific serine/threonine protein kinase (EC 2.7.11.1) |  |  |
| GL50803_9780 | Uncharacterized protein | GL50803_17254 | Phosphoglucomutase |  |  |
| GL50803_4652 | Ribosomal protein S16 | GL50803_103709 | BRO1-like domain-containing protein |  |  |
| GL50803_8217 | Uridine kinase | GL50803_9515 | Coiled-coil protein |  |  |
|  |  | GL50803_7188 | Uncharacterized protein |  |  |
|  |  | GL50803_12216 | Vacuolar ATP synthase subunit B |  |  |
|  |  | GL50803_3331 | malate dehydrogenase (EC 1.1.1.37) |  |  |
|  |  | GL50803_8044 | Seven transmembrane protein 1 |  |  |
|  |  | GL50803_40817 | Actin |  |  |
|  |  | GL50803_40224 | Transporter, MFS superfamily protein |  |  |
|  |  | GL50803_61564 | Ankyrin repeat protein 3 |  |  |
|  |  | GL50803_5883 | Uncharacterized protein |  |  |
|  |  | GL50803_7031 | Spindle pole protein |  |  |
|  |  | GL50803_86468 | Uncharacterized protein |  |  |
|  |  | GL50803_87519 | Uncharacterized protein |  |  |
|  |  | GL50803_9704 | Transketolase |  |  |
|  |  | GL50803_7195 | Glutamate synthase |  |  |
|  |  | GL50803_14311 | Serine/threonine-protein phosphatase (EC 3.1.3.16) |  |  |
|  |  | GL50803_11301 | nucleoside-diphosphate kinase (EC 2.7.4.6) |  |  |
|  |  | GL50803_6920 | Ankyrin repeat protein 1 |  |  |
|  |  | GL50803_95593 | non-specific serine/threonine protein kinase (EC 2.7.11.1) |  |  |
|  |  | GL50803_97219 | rRNA 2'-O-methyltransferase fibrillarin (Histone-glutamine methyltransferase) |  |  |
|  |  | GL50803_15871 | Serine peptidase |  |  |
|  |  | GL50803_9062 | Long chain fatty acid CoA ligase 5 |  |  |
|  |  | GL50803_30476 | Long chain fatty acid CoA ligase 4 |  |  |
|  |  | GL50803_13152 | Ankyrin repeat protein 1 |  |  |
|  |  | GL50803_16125 | FAD-dependent glycerol-3-phosphate dehydrogenase |  |  |
|  |  | GL50803_10843 | Serine carboxypeptidase |  |  |
|  |  | GL50803_113133 | Uncharacterized protein |  |  |
|  |  | GL50803_17143 | Pyruvate kinase (EC 2.7.1.40) |  |  |
|  |  | GL50803_7204 | RNA recognition motif family protein |  |  |
|  |  | GL50803_114777 | Major facilitator superfamily transporter |  |  |
|  |  | GL50803_5649 | Alpha-10 giardin |  |  |
|  |  | GL50803_24537 | Uncharacterized protein |  |  |
|  |  | GL50803_41512 | Flagella associated protein |  |  |
|  |  | GL50803_21444 | Spindle pole protein |  |  |
|  |  | GL50803_113677 | Coiled-coil protein |  |  |
|  |  | GL50803_14285 | Malate dehydrogenase |  |  |
|  |  | GL50803_17400 | Cyclin |  |  |
|  |  | GL50803_10255 | RNA helicase (EC 3.6.4.13) |  |  |
|  |  | GL50803_10623 | phosphoenolpyruvate carboxykinase (GTP) (EC 4.1.1.32) |  |  |
|  |  | GL50803_10167 | Uncharacterized protein |  |  |
|  |  | GL50803_9750 | Intraflagellar transport protein 74/72 (IFT74/72) (Intraflagellar transport protein IFT74/72) (Intraflagellar transport protein component IFT74/72) |  |  |
|  |  | GL50803_14742 | non-specific serine/threonine protein kinase (EC 2.7.11.1) |  |  |
|  |  | GL50803_26199 | non-specific serine/threonine protein kinase (EC 2.7.11.1) |  |  |
|  |  | GL50803_16648 | Uncharacterized protein |  |  |
|  |  | GL50803_7532 | H(+)-transporting two-sector ATPase (EC 7.1.2.2) |  |  |
|  |  | GL50803_112063 | Major facilitator superfamily transporter |  |  |
|  |  | GL50803_11720 | Ankyrin repeat protein 1 |  |  |
|  |  | GL50803_87926 | Uncharacterized protein |  |  |
|  |  | GL50803_24842 | Ankyrin repeat protein 1 |  |  |
|  |  | GL50803_113030 | Kinase, NEK |  |  |
|  |  | GL50803_16124 | T-complex protein 1 subunit eta (TCP-1-eta) (CCT-eta) |  |  |
|  |  | GL50803_15591 | Coiled-coil protein |  |  |
|  |  | GL50803_16160 | Cathepsin B |  |  |
|  |  | GL50803_16779 | Cathepsin B |  |  |
|  |  | GL50803_14019 | Cathepsin B |  |  |
|  |  | GL50803_21063 | RNA pol II accessory factor, Cdc73 family, C-terminal domain-containing protein |  |  |
|  |  | GL50803_6464 | Uncharacterized protein |  |  |
|  |  | GL50803_d40995 | IFT complex B |  |  |
|  |  | GL50803_6184 | Branched-chain amino acid aminotransferase |  |  |
|  |  | GL50803_9720 | Ankyrin repeat protein 1 |  |  |
|  |  | GL50803_16667 | Acyl-CoA synthetase |  |  |
|  |  | GL50803_10524 | Uncharacterized protein |  |  |
|  |  | GL50803_112304 | Elongation factor 1-alpha |  |  |
|  |  | GL50803_6687 | Glyceraldehyde-3-phosphate dehydrogenase (EC 1.2.1.12) |  |  |
|  |  | GL50803_9824 | proteasome endopeptidase complex (EC 3.4.25.1) |  |  |
